# Bridging Vibrations and Spins: Mode-Resolved Spin–Phonon Coupling Revealed through THz EPR/Magnetic IR Simulation

**DOI:** 10.1021/acs.jpca.5c07944

**Published:** 2026-02-10

**Authors:** Haowei Chen, Maurice van Gastel, Alexander Schnegg, Frank Neese

**Affiliations:** † Department of Molecular Theory and Spectroscopy, 28314 Max-Planck-Institut für Kohlenforschung, Kaiser Wilhelmplatz 1, 45470 Mülheim an der Ruhr Germany; ‡ EPR Research Group, 28313 Max-Planck-Institut für Chemische Energiekonversion, Stiftstrasse 34-36, 45470, Mülheim an der Ruhr, Germany

## Abstract

Molecular electron spin qubits show great potential for quantum information storage and processing but require paramagnetic systems with slow relaxation, where coherence is limited by spin–phonon coupling. Understanding this coupling remains challenging due to scarce observables and conflicting models, making direct experimental insights crucial. Vibrational spectroscopy (IR and Raman) under magnetic fields provides a promising approach. Here, we present the first comprehensive simulation protocol to extract spin–phonon coupling parameters from THz EPR/Magnetic IR spectra. Different spectral features are illustrated using a one-phonon model, emphasizing cases where the coupling is weaker than the line width. Using a tetrahedral high-spin Co­(II) complex, we validate the method and benchmark it against quantum-chemical calculations. The dominant coupling is attributed to a twisting mode of the first coordination sphere. Excellent agreement among experiment, simulation, and computation demonstrates the power of THz EPR/Magnetic IR spectroscopy as a direct probe of spin–phonon coupling.

## Graphical abstract

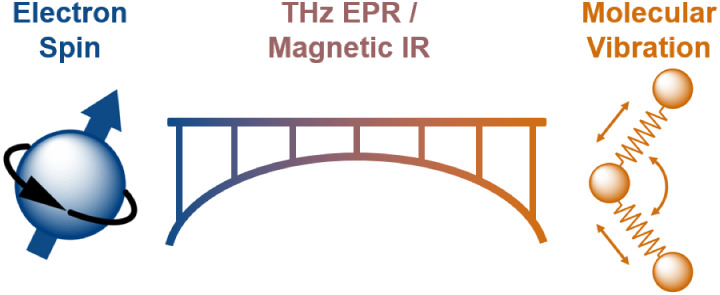

## Introduction

The concept of using paramagnetic transition metal complexes as molecular electron spin quantum bits (qubits) for information storage and processing has attracted considerable attention in recent years.^1–3^ To implement any quantum information protocol using such molecular qubits, it is essential to prepare spin states that can retain their orientation and phase coherence with high fidelity over extended period of time.^4–7^ Accordingly, transition metal complexes exhibiting slow magnetic relaxation are promising candidates.^8–22^

In spin-dilute environments, the phase memory time becomes increasingly limited at elevated temperatures by the spin–lattice relaxation time *T*_1_, which quantifies the rate at which energy of the spin system is dissipated into vibrational motions.^23,24^ Three primary spin–phonon relaxation mechanisms, direct, Orbach, and Raman processes, govern this relaxation, and all become increasingly detrimental to qubit coherence at higher temperatures.^24–26^ Thus, the interaction between spin states and molecular vibrational modes, the spin–phonon coupling, plays a central role in determining the coherence properties of molecular spin qubits.^7,27,28^ In addition, secondary processes may oftentimes also have a profound influence as well. For example, it is well-known in EPR spectroscopy that H/D exchange of either the surrounding solvent or host, or of the target system itself can significantly shorten the *T*_1_ time,^29,30^ providing experimental evidence that spin–lattice relaxation is achieved via the nuclear spin system that couples to both the electron spin and the phonons and as such serves as a mediator.

As such, although an explicit relation between spin–phonon coupling and *T*_1_ to the best of our knowledge has not been established based on the multiple processes that contribute, for the direct processes, it raises a fundamental question: which vibrational modes most effectively induce spin–phonon coupling, and how strong is this coupling? Numerous theoretical studies have sought to predict and understand this phenomenon.^7,28,31–41^ However, validating these models typically requires either high-level calculations or comparison with experimental data. From the experimental perspective, spin relaxation rates are often used as benchmarks, but these require a theoretical framework that accounts for all relevant relaxation mechanisms simultaneously. As a result, discrepancies between theory and experiment are common, and quantitative agreement remains rare.^40^ This ambiguity makes it difficult to determine whether observed deviations stem from inaccurate modeling of spin–phonon coupling itself, or from limitations in how the relaxation mechanisms are treated.

To resolve this, more direct experimental insights into spin–phonon coupling are needed. A promising approach is to probe vibrational spectroscopy, such as IR or Raman, under an external magnetic field.^5,19,42–62^ Experimentally, two principal configurations are typically used for the relative orientation of the magnetic field to the propagation vector (*k⃗*) of the incident light: when the field (*B⃗*_0_) is aligned parallel to *k⃗*, the setup is referred to as the Faraday mode, whereas when the field is oriented perpendicular to *k⃗*, it is termed the Voigt mode. In such experiments, magnetic fields induce shifts of the magnetic sublevels; if spin–phonon coupling is operative, these shifts can lead to interactions between spin and vibrational transitions, which would manifest as avoided crossings in the spectra. Though such direct observations of spin–phonon couplings are challenging, magnetic IR (also referred to as THz-EPR) spectroscopy has demonstrated interference features between spin and phonon absorptions. However, so far, simulations of these effects have typically relied on simplified energy-level diagrams and have omitted transition intensities or detailed light–matter interactions.^50,51,55,56,63^ While these models seem to qualitatively capture the field dependence, they provide limited insight into the redistribution of intensity between spin and phonon transitions, scarcely even casting doubt on the reliability of the extracted spin–phonon coupling parameters.

The goal of the present work is to establish a comprehensive simulation protocol to experimentally extract spin–phonon coupling information from magnetic IR/THz EPR spectra. We systematically analyze how various spin–phonon coupling parameters influence the spectral features and test the validity of our approach using an exemplary tetrahedral model complex, **1** (shown in Scheme 1), that has been recently investigated by THz EPR and features a high-spin Co^2+^ ion, *S* = 3/2 with an axial zero-field splitting (ZFS) parameter *D* = −23.0 cm^–1^. The reason for choosing this *S* = 3/2 system as a prototypical system as opposed to *S* = 1/2 systems is that by the ZFS it provides a large enough frequency offset of the EPR signal that it becomes detectable in the far-infrared region by THz EPR spectroscopy. We also note that the relative contributions of the aforementioned relaxation mechanisms will depend on the frequency region in which they occur and as such may be different for *S* = 1/2 systems as compared to *S* > 1/2 systems.

**Scheme 1:**
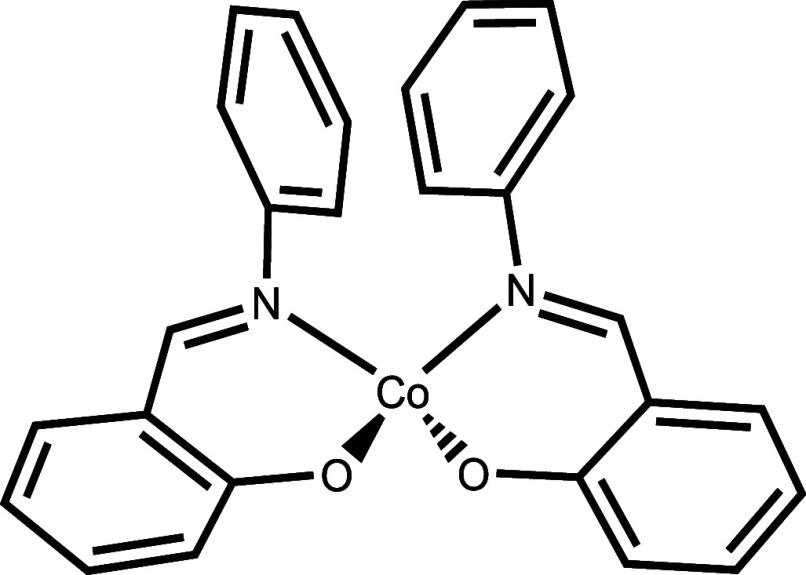
Molecular Structure of **1**

## Methods

All calculations were carried out with the ORCA program package (version 6.1).^64^ Three systems were examined: two model systems [CoCl_4_]^2–^ and [Co­(ndh)]_2_]^2–^, and complex **1**. Geometry optimizations were performed at TPSSh/def2-TZVP level without solvation model,^65,66^ employing Grimme’s D3BJ dispersion correction,^67,68^ the RIJCOSX approximation,^69–71^ and ORCA’s default auxiliary basis sets^72^ and convergence criteria.

Single-point calculations were first performed using the Complete Active Space Self Consistent Field and N-electron Valence Space Perturbation theory (CASSCF/NEVPT2) framework^73–75^ with a CAS­(7,5) active space (five 3d orbitals, seven electrons). Ten quartet and 40 doublets were first included in the state-average calculation, followed by quasi-degenerate perturbation theory (QDPT) for spin–orbit coupling (SOC) and magnetic properties. As the doublets contributed negligibly to the magnetic properties, subsequent calculations included only ten quartets. Scalar relativistic effects were treated with the X2C Hamiltonian and x2c-TZVPall basis,^76–79^ including second-order picture-change corrections, though results were essentially identical to the nonrelativistic treatment.

Ab initio ligand field theory (AILFT)^80,81^ defines five d orbital based on the *x*, *y*, *z* axes of the Cartesian axes. Therefore, we first rotate the molecular coordinates to best match and purify the representation under AILFT framework:

1 In [CoCl_4_]^2–^, *x*, *y*, and *z* aligned with three *S*_4_ axes.
2 In [Co­(ndh)]_2_]^2–^, *z* was aligned with the *S*_4_ axis, and *x*, *y* with the two perpendicular *C*_2_ axes.
3 In complex **1**, *x*, *y*, and *z* were aligned with the principal axes of the *D*-tensor at the equilibrium structure.

Frequency calculations were performed at the TPSSh/def2-TZVP level, and the resulting Hessian, vibrational modes, and frequencies were used for spin–phonon coupling analysis. The symmetry constraints were applied in the [CoCl_4_]^2–^ model, so that degenerate E and T_2_ vibrational modes do not mix randomly.

Vibrational modes were first visualized and those related to motion in the first-coordination sphere were selected. For each vibrational mode selected, displacements along the normal coordinate up to ±0.5 Å were applied in 0.05 Å increments on the equilibrium structure, yielding 21 geometries per mode. For each displaced structure, CASSCF/NEVPT2 single-point calculations were performed with the setup described above to extract spin Hamiltonian parameters. The resulting AILFT outputs and *D*-tensors were examined and the latter showed negligible rotation of the principal axes; thus, the ZFS parameters *D* and *E* were plotted as functions of the normal coordinates. A third-order polynomial fit was then applied, and the linear term was taken as the spin–phonon coupling parameter.

## Results

### THz EPR Measurements of **1**

Voigt-mode THz EPR measurements have been performed at *T* = 5 K and from 0 T to 7 T in steps of 0.5 T on a **1**-polyethylene pellet. This data set has been published previously by Pohle et al.^55^

The raw transmission spectra of **1** are presented in Figure 1 (left). These spectra represent the cumulative contribution of the emission spectrum of the light source, sample, the absorption in the transmission line the sample and the energy dependent detector characteristics. A field-dependent feature appears near 47 cm^–1^ (circled in orange), superimposed on an oscillatory background. The latter originates from interference due to reflections between the parallel surfaces of the pellet. To isolate intrinsic FIR spectral features from this oscillatory background, a Fourier transformation combined with high-frequency filtering was applied. As demonstrated in Figure S2, this process effectively suppresses the interference pattern without introducing or removing genuine spectral signals. In addition to the main field-dependent feature, two absorption-like features are observed at 45.8 cm^–1^ and 54.8 cm^–1^ (denoted by vertical gray dashed lines). As the magnetic field is increased from 0 to 7 T, the field dependent feature decreases slightly in intensity at about 47 cm^–1^, while the absorption-like feature, especially the one at 54.8 cm^–1^, remains almost unchanged. Since no such absorption features in other THz spectra with the same setup are observed, they arise from absorption of the sample. Therefore, we assign the field dependent feature to near 47 cm^–1^ to an EPR transition and those at 45.8 cm^–1^ and 54.8 cm^–1^ as low-frequency vibrational bands, i.e., phonons.

**Figure 1:**
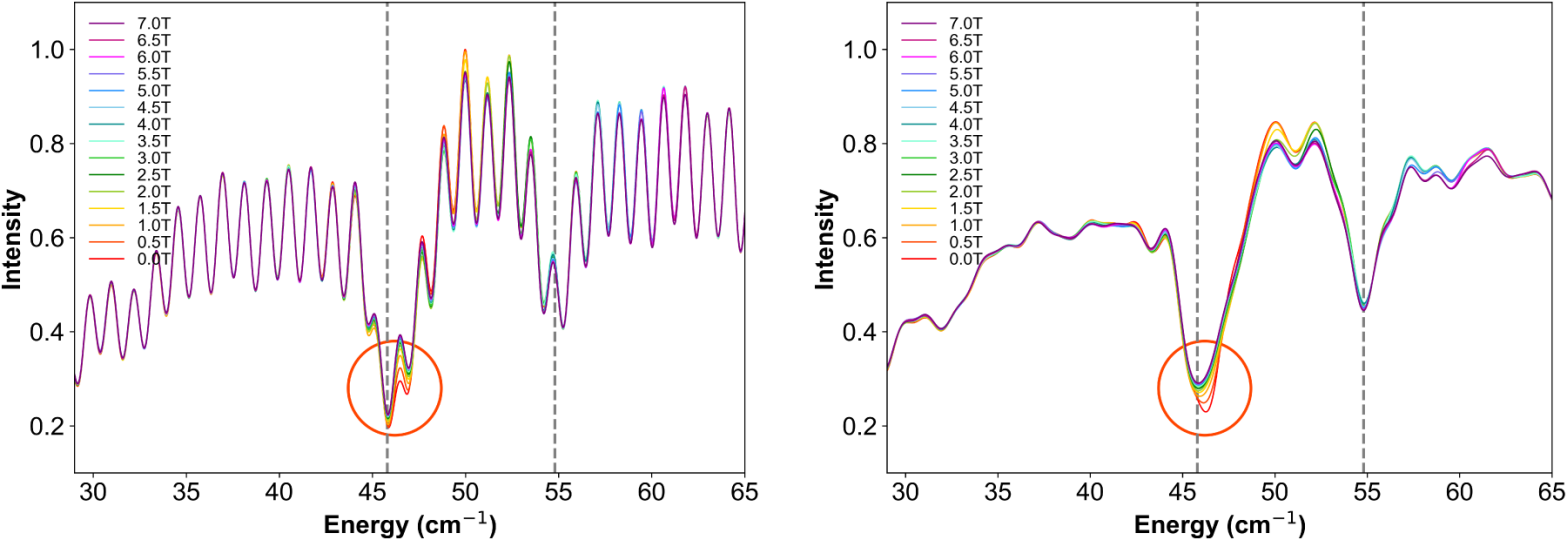
(left) THz EPR raw transmission data of a pellet of complex **1** from 0 T to 7 T, measured at *T* = 5 K and in Voigt mode. (right) Transmission data after filtering the high-frequency oscillation.

Allowed vibrational transitions are typically electric-dipole driven and much stronger than EPR transitions (typically 4 orders of magnitude stronger), which are magnetic-dipole in nature and field-dependent. In this case, though the phonons have higher intensity than the field-dependent features, their relative intensities are comparable. In order to display the field dependence in a more pronounced manner, the spectra are typically shown as division spectra.^82^ For a field progression of THz EPR data, the background spectrum for a certain magnetic field is typically chosen to be the transmission spectrum at lower magnetic field. As such, the field progression is made clear by a division-progression series that starts at 0.5 T/0 T and ends at 7.0 T/6.5 T, where the transmission data of the spectrum and the background at 0.5 T lower field are divided on a point-by-point basis. The division spectra are shown in Figure 2(a). The procedure effectively removes the oscillatory background—even in the absence of signal filtration—as well as the field-independent absorption-like contributions in the spectrum.

**Figure 2:**
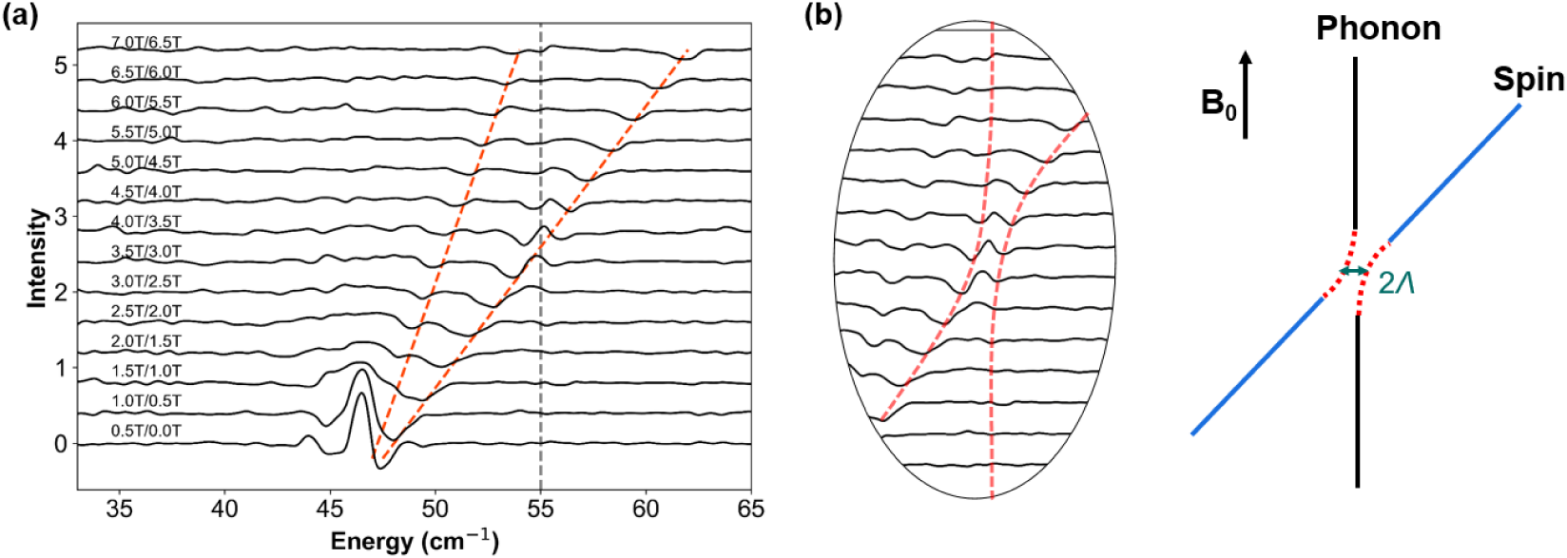
(a) THz EPR magnetic field division spectra of a pellet of complex **1** from 0 T to 7 T, measured at *T* = 5 K and in Voigt mode ranging from 0.5 T/0 T to 7.0 T/6.5 T. The orange dashed line denotes the spectral branch evolution, the gray dashed line denotes absorption-like feature at 54.8 cm^–1^. (b) A zoom-in of the avoided crossing feature (the second derivative-like shape) around 54.8 cm^–1^, where Λ denotes the phenomenological coupling strength between the spin and phonon states.

The division spectra more clearly reveal field-dependent features that are obscured in the transmission spectra. Upon elevating the magnetic field from 0 to 7 T, the field-dependent feature exhibits a pronounced spectral evolution, one distinct branch shifts upward from 47 cm^–1^ to 62 cm^–1^, with a weak branch shifts upward from 47 cm^–1^ to 54 cm^–1^. The upshift branching behavior is distinctly different from conventional THz EPR spectra,^82^ as it exhibits pronounced second-derivative w-shaped “wiggle” structures when the branch crosses the phonon position as shown in Figure 2(b). Such structure is indicative of spin–phonon coupling,^51,56^ and therefore a systematic framework needs to be established to enable quantitative modeling and extraction of the enclosed information. This approach is described in the following section.

### Extended Effective Hamiltonian

The underlying logic of our approach is straightforward: first we construct a description of the system’s energy levels and explicitly incorporate spin–phonon coupling. Second, we formulate the light–matter interaction to quantitatively describe the transitions between these states, thereby enabling a quantitative interpretation of the spectral features. Since experiments have been performed at low temperature and in the low-frequency region, vibrational overtones and electronic excitations generally do not play a role. It therefore suffices to consider the vibrational and spin sublevels in the ground electronic state. As such we confine ourselves to a subspace with reduced dimensionality and aim to use an effective Hamiltonian to reproduce the property in this subspace. In particular, we use a direct product basis set to describe the system:

1 $$\left|v,SM_{S}\right\rangle =|v\rangle ⊗|SM_{S}\rangle$$

2 $$|v\rangle =\left\{n_{1}...n_{N}\right\}$$

Here, the vibrational wave function is expressed in the form of a vector, in which the set of vibrational quanta {*n*_1_···*n*_*N*_} enumerates the vibrational levels of in total *N* phonons considered in the treatment. Each vibrational quantum *n*_α_ is limited to be either 0, corresponding to the vibrational ground state or 1, corresponding to the first vibrational excited state. Lastly, *S* indicates the spin multiplicity and the *M_S_* quantum number enumerates the magnetic sublevels with .*M_S_* = *S, S*–1, ···, *–S* As such, the dimension of this basis set equals 2^N^(2*S* + 1). For practical purposes, the maximum number of phonons considered in the current implementation is 8.

With this basis set, we select an extended spin Hamiltonian as the effective Hamiltonian to describe the energy level of the system. It consists of a conventional spin Hamiltonian, a harmonic oscillator description of the phonon, and the spin–phonon coupling.

3 $${Ĥ}_{\mathrm{tot}}={Ĥ}_{S}+{Ĥ}_{\mathrm{Ph}}+{Ĥ}_{S-\mathrm{Ph}}$$

The conventional spin Hamiltonian part is the form that has been widely used in EPR spectroscopy^83,84^ and is given by

4 $${Ĥ}_{S}=\mu_{B}{\mathrm{B⃗}}_{0}\cdot g\cdot Ŝ+Ŝ\cdot D\cdot Ŝ$$

where μ_B_ is the Bohr magneton, ***g*** is the g-tensor and ***D*** is the ZFS-tensor. This Hamiltonian gives rise to contributions that are block-diagonal in all vibrational quanta *n*_α_; it also implies that the spin-Hamiltonians are the same for different vibrational quanta.

The second term in the extended effective Hamiltonian provides the energies associated with the phonons and is given by the energy of a quantum mechanical harmonic oscillator:

5 $${Ĥ}_{\mathrm{Ph}}=\overset{N}{\underset{\alpha }{\sum }}\hbar \omega_{\alpha }\left({\mathrm{n̂}}_{\alpha }+\frac{1}{2}\right)$$

ω_α_ and *n̂*_α_ are the vibrational frequency and quanta operator of the α-th phonon. Summation of *Ĥ*_S_ and *Ĥ*_Ph_ leads to the uncoupled Hamiltonian that, in matrix form, is block diagonal in the vibrational quanta as shown in Supporting Information.

The third term describes the coupling between spin and vibrational levels. The nature of the spin–phonon coupling is considered as how (molecular) vibrations modulate the magnetic sublevels. To this end, it is customary to begin with the approximation that the electronic spin system is initially not coupled to the phonons. The spin–phonon coupling is then introduced as a perturbation via a Taylor expansion of the uncoupled zeroth-order Hamiltonian with respect to each normal mode.^27,85^ For the purpose of spectral simulation, we adopt the conventional spin Hamiltonian as the zeroth-order Hamiltonian. Accordingly, the spin–phonon coupling Hamiltonian *Ĥ*_S–Ph_ is defined as

6 $${Ĥ}_{S-\mathrm{Ph}}=\overset{N}{\underset{\alpha }{\sum }}\left(\frac{\partial {Ĥ}_{S}}{\partial Q_{\alpha }}\right){\mathrm{Q̂}}_{\alpha }+\frac{1}{2}\overset{N}{\underset{\alpha ,\beta }{\sum }}\left(\frac{\partial^{2}{Ĥ}_{S}}{\partial Q_{\alpha }\partial Q_{\beta }}\right){\mathrm{Q̂}}_{\alpha }{\mathrm{Q̂}}_{\beta }+\cdot \cdot \cdot$$

where *Q*_α_ is the normal coordinate of the α-th phonon.

The first-order term, the linear term, couples the magnetic sublevels of one phonon. The second-order term, has no contribution from a single phonon under the Harmonic oscillator approximation, rather it couples magnetic sublevels with two phonons. The introduction of the second-order term would in addition introduce a quadratic number of spin–phonon coupling parameters, which would lead to overfitting of the present experimental spectra. Therefore, we limit ourselves to the linear term of the spin–phonon coupling Hamiltonian which becomes in general form:

7 $${Ĥ}_{S-\mathrm{Ph}}=\overset{N}{\underset{\alpha }{\sum }}\left(\mu_{B}{\mathrm{B⃗}}_{0}\cdot \left(\frac{\partial g}{\partial Q_{\alpha }}\right)\cdot Ŝ+Ŝ\cdot \left(\frac{\partial D}{\partial Q_{\alpha }}\right)\cdot Ŝ\right){\mathrm{Q̂}}_{\alpha }$$

However, the full parameter set introduces a large number of variables, which can easily lead to overfitting and render the physical interpretation ambiguous. To minimize parameter redundancy, we impose constraints on the formalism by adopting the form that mirrors the commonly used spin Hamiltonian, in which ***g*** and ***D*** are collinear. Specifically, in the interest of simplicity we assume that the principal axes of these tensors remain unchanged to first order under vibrational displacements. Thus, we use the spin Hamiltonian and spin–phonon coupling of:

8 $${Ĥ}_{S}=\underset{i=x,y,z}{\sum }\mu_{B}B_{0}l_{i}g_{i}{Ŝ}_{i}+D\left({Ŝ}_{z}^{2}-\frac{1}{3}S\left(S+1\right)\right)+E\left({Ŝ}_{x}^{2}-{Ŝ}_{y}^{2}\right)$$

9 $${Ĥ}_{S-\mathrm{Ph}}=\overset{N}{\underset{\alpha }{\sum }}\left[\underset{i=x,y,z}{\sum }\mu_{B}B_{0}l_{i}\left(\frac{\partial g_{i}}{\partial Q_{\alpha }}\right){Ŝ}_{i}+\left(\frac{\partial D}{\partial Q_{\alpha }}\right)\left({Ŝ}_{z}^{2}-\frac{1}{3}S\left(S+1\right)\right)+\left(\frac{\partial E}{\partial Q_{\alpha }}\right)\left({Ŝ}_{x}^{2}-{Ŝ}_{y}^{2}\right)\right]{\mathrm{Q̂}}_{\alpha }$$

where *l⃗* being a dimensionless unit vector, its projection to *i*-direction is *l*_*i*_.

This form of the spin-Hamiltonian significantly reduces the number of independent spin–phonon coupling parameters per vibrational mode, from 11 to 5. For each vibrational mode, this gives us spin–phonon coupling parameters $\frac{\partial g_{i}}{\partial Q_{\alpha }}$ , $\frac{\partial D}{\partial Q_{\alpha }}$ , and $\frac{\partial E}{\partial Q_{\alpha }}$ that describe the modulation of the spin-Hamiltonian parameters *g_i_*, *D*, and *E* along the normal coordinate *Q*_α_. For simplicity, we use $g_{i}^{\alpha }$ , *D* ^α^, and *E* ^α^ to represent the spin–phonon coupling parameters (for more details, please refer to the Supporting Information).

### Interaction with the THz Field

The final ingredient required for the simulation is the light–matter interaction, specifically the interaction between the system and the THz radiation. In conventional IR spectroscopy, vibrational transitions are typically electric-dipole based, which requires a change in the electric dipole moment. They are induced by the electric field component of the radiation. In contrast, EPR spectroscopy involves spin-flip transitions, which are induced by the magnetic field component of the radiation. It is expected that the spin-flip transitions are much weaker than the vibrational transitions, but given that both spin-flip and vibrational transitions are relevant in our system, it is necessary to consider the system’s interaction with both the electric and magnetic components of the radiation field. Therefore, the light–matter interaction Hamiltonian *Ĥ*_1_(*t*) is constructed as

10 Ĥ1(t)=−E⃗1⁡cos(ωt)·μ⃗^+μBB⃗1⁡cos(ωt)·g·Ŝ

ω is the radiation frequency, *E⃗*_1_ cos­(ω*t*) and *B⃗*_1_ cos­(ω*t*) are the electric and magnetic field components of the electromagnetic wave, respectively. The radiation propagates along *k⃗*, and *k⃗*, *E⃗*_1_ and *B⃗*_1_ are mutually orthogonal. We then apply Fermi’s golden rule to calculate the transition probabilities between different eigenstates of the extended spin Hamiltonian.

11 $$P_{\mathrm{If}}\left(t\right)=\frac{2\pi }{\hbar }{|\langle \Psi_{I}|{Ĥ}_{1}\left(t\right)|\Psi_{F}\rangle |}^{2}$$

Here, Ψ_I_ and Ψ_F_ denote the initial and final states, respectively, which are the eigenstates of the extended spin Hamiltonian *Ĥ*_S_ + *Ĥ*_Ph_ + *Ĥ*_S–Ph_, expressed as linear combinations of functions of our direct product basis. For *Ĥ*_1_(*t*) we have presently implemented a Faraday mode variation where *k⃗* ∥ *B⃗*_0_ and a Voigt mode variation where *k⃗* ⊥ *B⃗*_0_ for unpolarized THz radiation. We believe that Fermi’s golden rule is sufficient for the THz EPR experiment, since it is sensitive to (instantaneous) 1-photon absorption and not to follow-up relaxation processes. Moreover, the THz radiation field is very weak such that excited-state populations are negligible and two-photon processes are unlikely. We can therefore safely neglect any time-dependent processes following the initial absorption event as the experiment is not sensitive to them and treat the ground-state population as quasi-stationary.

### Description of Phonon Transitions and Spin–Phonon Coupling Parameters

In practical simulations, we do not calculate the transition electric dipole moment by explicitly integrating over vibrational wave functions. Rather, we describe it by assigning an effective transition dipole moment vector. Thus, each phonon principally is described by the following properties: (1) the frequency at which the phonon occurs, (2) the relative amplitude of the transition electric dipole moment, (3) the orientation of the transition electric dipole moment, and (4) the line width. The third property is described by two Euler angles, using the principal axes of the ZFS tensor as a reference system. In total, this amounts to 5 parameters per phonon.

In the matrix form, the light–matter interaction is given by

12 $${\left({Ĥ}_{1}\right)}_{v,M_{S},v^{\prime },{M_{S}}^{\prime }}=-{\mathrm{E⃗}}_{1}\cdot \delta_{M_{S}{M_{S}}^{\prime }}\left(\overset{N}{\underset{\alpha =1}{\sum }}\delta_{...n_{\alpha }...,...{n_{\alpha }}^{\prime }\pm 1...}{\mathrm{\mu ⃗}}_{\alpha }\right)+\mu_{B}\delta_{vv^{\prime }}\langle vM_{S}|{\mathrm{B⃗}}_{1}\cdot g\cdot Ŝ\left|v^{\prime }{M_{S}}^{\prime }\right\rangle$$

where two angles specify the direction of the electric transition dipole μ⃗_α_ with respect to the ZFS principal axes system. The first term *E⃗*_1_·μ⃗_α_ couples the electric field with the transition electric dipole moment operating between ground and first excited vibrational levels of α-th vibrational mode, while the second term represents the EPR or spin-flip transition. For clarity, we refer to transitions driven by the spin-free electric dipole operator as vibrational transitions or phonons, whereas those driven by the spin-dependent magnetic dipole operator correspond to EPR or spin-flip transitions. Electric-dipole-allowed transitions are generally more intense than magnetic-dipole transitions by roughly 4 orders of magnitude; the relative amplitude $\frac{\left|{\mathrm{\mu ⃗}}_{\alpha }\right|\left|{\mathrm{E⃗}}_{1}\right|}{\mu_{B}\left|{\mathrm{B⃗}}_{1}\right|}$ of the electric dipole and the EPR transition, which is dimensionless, is specified as the relative intensity parameter in simulations. Within the present definition of light–matter interaction, no operator can directly induce vibrational transitions accompanied by a spin flip; such processes arise exclusively through spin–phonon coupling, which mixes the magnetic and vibrational wave functions.

In general, nondegenerate normal modes are only determined up to an arbitrary phase factor. Consequently, the sign of the spin–phonon coupling parameter is inherently depended on that phase. Thus, multiplying the normal mode by −1 not only inverts the sign of the corresponding spin–phonon coupling parameter but also alters the sign of the associated phonon transition dipole moment. This thought experiment highlights that the *relative sign*, or phase, between the spin–phonon coupling parameter and the phonon transition dipole moment carries physical significance and directly impacts the outcome of spectral simulations.

### Powder Spectra

For powder spectra, where the magnetic property is potentially highly anisotropic, the simulations have to be performed for many different orientations uniformly distributed over the space. For this purpose, we construct a grid of equidistant points and a weight proportional to an associated trapezoid-shaped surface element so that the entire unit sphere is covered. We then summed the “single-crystal spectra” from all the calculated orientations, using the principal axes system of the D tensor as a reference system. In practice, integration over 1/8th of the unit sphere (θ = (0, π/2), φ = (0, π/2)) suffices.

## Discussion

### Simulation of a One-Phonon Model

In this section, a simple one-phonon model is employed to illustrate the simulation procedure and to demonstrate the impact of spin–phonon coupling on the field-division spectra. We begin with an *S* = 3/2 spin system with a large positive *D*-value, and then sequentially incorporate the phonon and spin–phonon coupling to examine the resulting spectral features.

Assuming the academic *S* = 3/2 system has a positive *D*-value of 45 cm^–1^ and no rhombicity (*E*/*D* = 0), with an isotropic *g*-tensor (*g*_*x*_ *= g*_*y*_ *= g*_*z*_ = *g*_iso_ = 2), and initially no phonon is present, the corresponding energy levels under an external magnetic field are shown in Figure 3(a). In this model, the system can only undergo a spin-flip transition. The orientation-average powder spectrum at 10 T is depicted in Figure 3(b). At low temperature and high-field, the thermal population is predominantly in the lowest magnetic sublevel, and thus only two transitions—specifically, the 1–3 and 1–4 lines (from the ground sublevel to the third and fourth sublevels)—are relevant in the region of interest. The turning points of the absorption peaks correspond to the 1–3 and 1–4 lines with *B* parallel to *z* and *B* parallel to *x*, *y*, respectively. Figure 3(c) also presents the transmission spectra as the magnetic field increases incrementally from 0 to 14 T in 1 T steps. At 0 T, the 1–3 and 1–4 lines coincide due to Kramers degeneracy, corresponding to the zero-field splitting (2*D*) of 90 cm^–1^; as the field increases, the magnetic anisotropy emerges, leading to a broadening of the spectrum over a wider range of frequencies. The corresponding field-division spectra are shown Figure 3(d). Upon elevating the field, the turning points in the transmission spectra, corresponding to the 1–3 and 1–4 lines with *B*∥*z* and *B*∥*x*,*y*, shift upward or downward and leave two clear spectral branches in the absorption and transmission spectra. As such, this procedure is identical to the one widely applied in simulating THz EPR spectra of high spin transition metal systems.^82^

**Figure 3:**
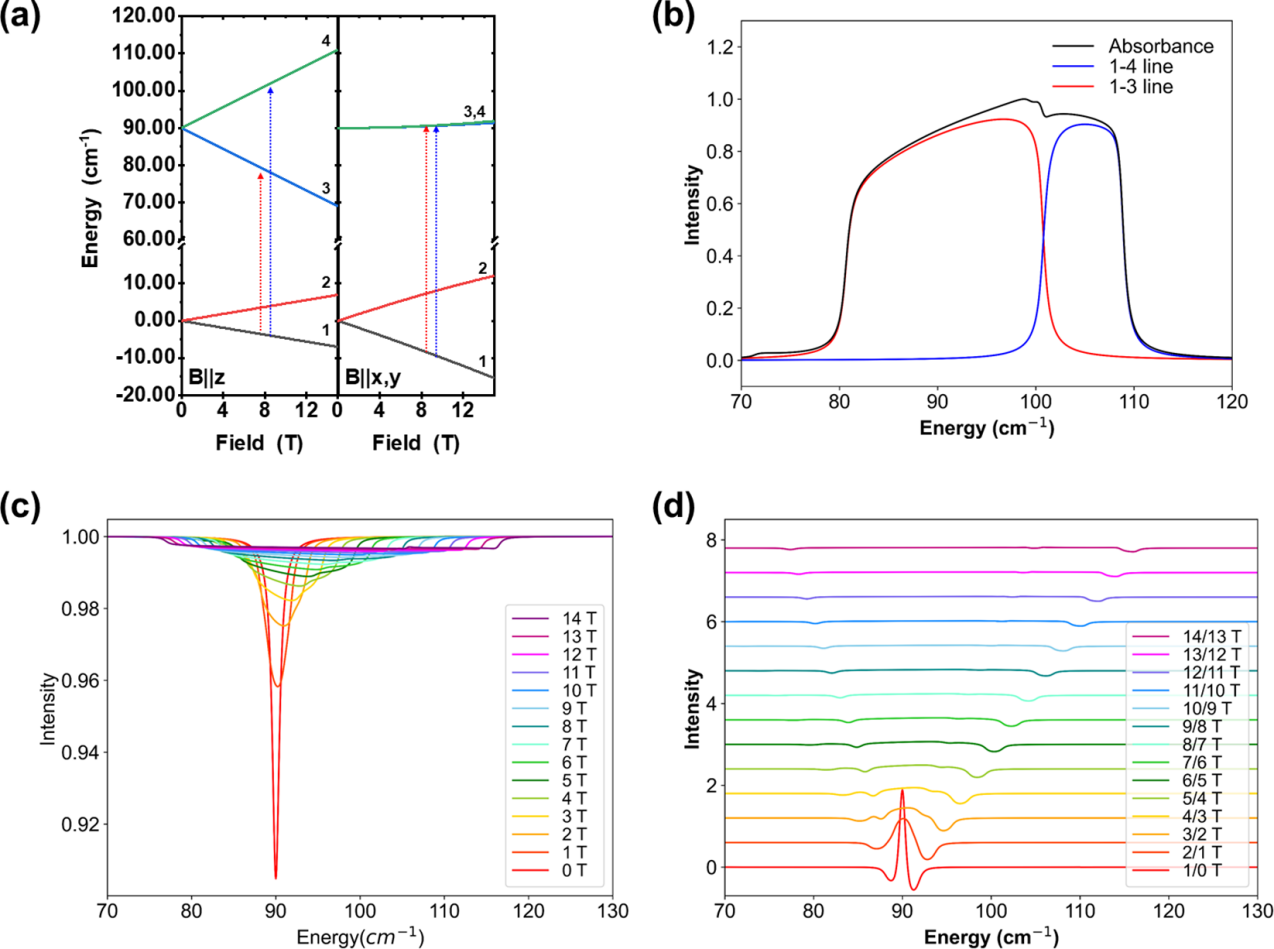
(a) Energy level diagram of the *S* = 3/2 system, *D* = 45 cm^–1^, *E*/*D* = 0, *g*_iso_ = 2. (b) Orientation averaged absorption spectrum at 10 T. (c) Orientation averaged transmission spectra upon elevating the field from 0 to 14 T. (d) Field-division spectra.

We then introduce a phonon at 100 cm^–1^, i.e., 10 cm^–1^ above the zero-field splitting (2*D*), and *x*-polarized. Assuming no spin–phonon coupling, the energy level diagram is shown in Figure 4(a). Typically, phonon absorptions are electric-dipole-based and much stronger than the spin-flip transitions. Therefore, we introduce an intense phonon alongside to the weaker EPR transition in the transmission spectra (shown in Figure 4(b)). Since there is no spin–phonon coupling, no mixing between the magnetic sublevels of the ground and first excited vibrational state occurs. Consequently, the field-division spectra (shown in Figure 4(c)) remain the same as without the phonon. In particular, no additional structure can be observed when the field-dependent branches cross the phonon.

**Figure 4:**
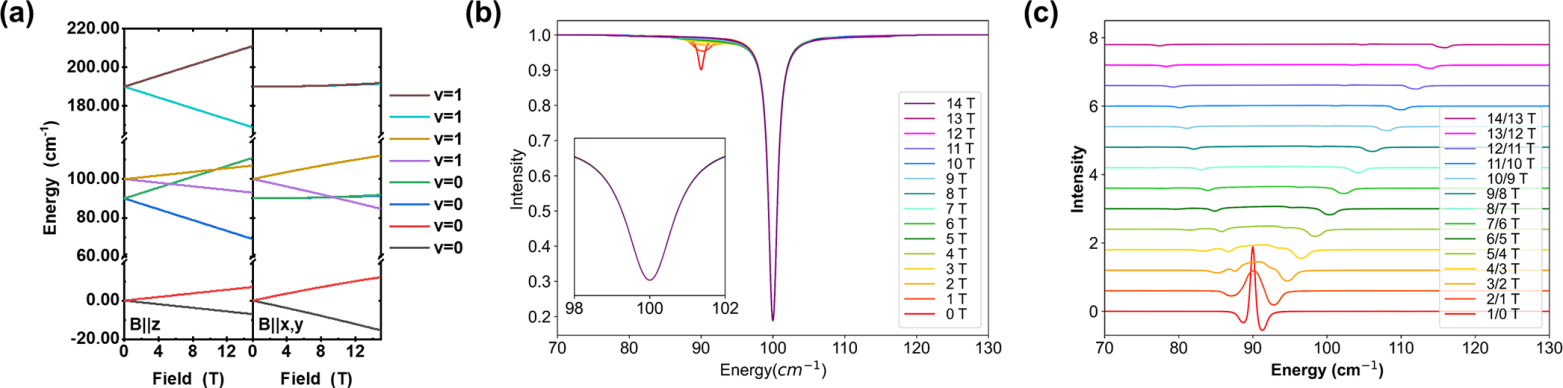
(a) Energy level diagram of the *S* = 3/2 system, *D* = 45 cm^–1^, *E*/*D* = 0, *g*_iso_ = 2, with one phonon absorption at 100 cm^–1^. (b) The transmission spectra upon elevating the field from 0 to 14 T. (c) The field-division spectrum.

Next, we introduce spin–phonon coupling to this system. For this specific phonon, the relevant spin–phonon coupling parameters include $g_{i}^{\alpha }$ , *D* ^α^, and *E* ^α^, yielding five independent parameters in total. Two central questions arise: how does each spin–phonon coupling parameter influence the spectra, and which among them exerts the most significant impact on the spectral features? The energy level diagram shown in Figure 4(a) actually already provides a hint. As the field increases, the *M*_*S*_ = +3/2 level of the vibrational ground state crosses the *M*_*S*_ = ±1/2 levels of the first vibrational excited state. One thus expects that the spin–phonon coupling is able to directly couple these two states that differ by 1 or 2 in their *M*_*S*_ values and to cause efficient mixing. By examining the off-diagonal spin–phonon coupling block of the Hamilton matrix (shown in Figure 5), it becomes evident that $g_{x/y}^{\alpha }$ and *E* ^α^ are primarily responsible for the interaction between these levels. Owing to historical precedence, prior studies have focused on the determination of *E* ^α^ by simulating the energy-level diagrams based on the observed avoided crossing in the division spectra of magnetic levels that differ by Δ*M*_*S*_ = ±2.^50,51,56,63^ Therefore, we begin our analysis with this spin–phonon coupling parameter. Subsequently, we consider $g_{x}^{\alpha }$ and $g_{y}^{\alpha }$ , which introduce off-diagonal matrix elements capable of directly coupling levels with Δ*M*_*S*_ = ±1. Finally, we examine $g_{z}^{\alpha }$ and *D* ^α^, whose contributions are expected to be minor, as they enter as diagonal elements in the spin–phonon coupling matrix and, thus, cannot efficiently mediate transitions between different *M*_*S*_ levels.

**Figure 5:**
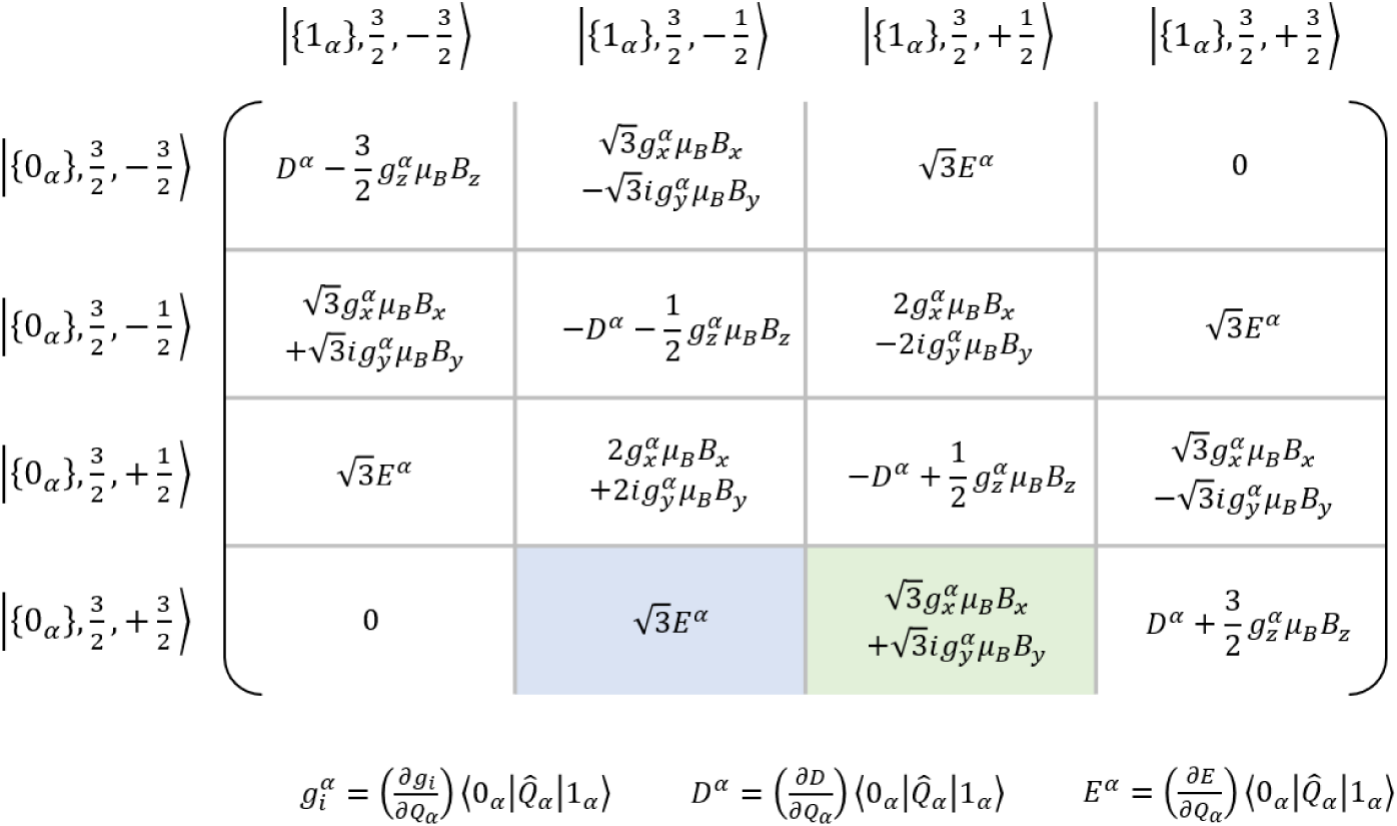
Spin–phonon coupling matrix of the *S* = 3/2 system with one phonon *α*.

### The Contribution of *E* ^α^

*E* ^α^ is primarily responsible for the interaction between *M*_*S*_ = +3/2 and *M*_*S*_ = −1/2 levels, since it is multiplied with the operator $\left({Ŝ}_{x}^{2}-{Ŝ}_{y}^{2}\right)$ . Inclusion of this spin–phonon coupling term leads to level mixing and to an avoided crossing in the energy level diagram, as illustrated in Figure 6(a), which was also the basis of energy level simulations in previous studies. Literature precedents exist that estimate this coupling strength directly from the field-division spectra, typically assigning this coupling strength on the order of 1 cm^–1^.^50,51,56,63^ Based on this, we assign the parameter *E* ^α^ a value of $1/\sqrt{3}$ cm^–1^ for testing purposes, resulting in the off-diagonal matrix element Λ_S–Ph_ of 1 cm^–1^ and an avoided crossing with a 2 cm^–1^ separation. The corresponding transmission and field-division spectra are shown in Figure 6(b) and (c), respectively. A characteristic second-derivative-like wiggle structure is observed when the upward shifting branch crosses the phonon. With an avoided crossing of 2 cm^–1^, the second-derivative-like structure is very intense, and moreover, the phonon even splits in the transmission spectra (Figure 6(b), inset). However, such a splitting has not been observed in any transmission data of THz EPR experiments. This constitutes a first fundamental question: what happens to the division spectra if the spin phonon-coupling is smaller than the line width?

**Figure 6:**
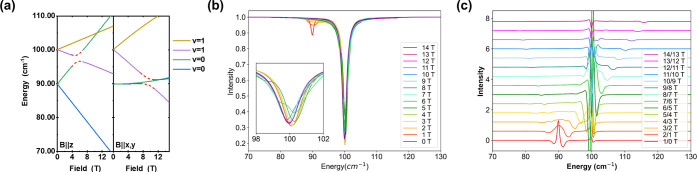
(a) Energy level diagram, *D* = 45 cm^–1^, *E*/*D* = 0, *g*_iso_ = 2, with one phonon at 100 cm^–1^, and *E* ^α^ of $1/\sqrt{3}$ cm^–1^. Only the avoided crossing region are shown. (b) Field-dependent transmission spectra from 0 to 14 T. (c) Field-division spectra.

### Division Spectra with Spin–Phonon Coupling Smaller Than Line Width

In matrix form using the | ***v***, *SM_S_* ⟩ basis set, the off-diagonal spin–phonon coupling matrix element Λ_S–Ph_, in this case $\sqrt{3}E^{\alpha }$ , couples two close-lying magnetic levels with different vibrational quantum numbers:

13 $$\left(\begin{array}{ll} \varepsilon_{n_{\alpha }=1,M_{S}=-1/2}\left(B\right) & \Lambda_{S-Ph}\\ \Lambda_{S-Ph} & \varepsilon_{n_{\alpha }=0,M_{S}=+3/2}\left(B\right) \end{array}\right)$$

It is important to emphasize that this off-diagonal matrix element can only lead to efficient level mixing and concomitant intensity borrowing when the difference of two diagonal elements is close to or less than 2Λ_S–Ph_. It is then common procedure to estimate the magnitude of the spin–phonon coupling constant from the spectrum by determining the distance of minimum approach of these levels. There is, however an important caveat to this approach one has to be aware of: in the division spectra, this distance does not directly reflect the magnitude of spin–phonon coupling, especially when the coupling strength is smaller than the spectral line width!

In order to illustrate what happens to avoided crossings in division spectra when the coupling is smaller than the line width, we present the following mathematical model: we assume two transitions of equal intensity and line width, with one shifted to higher energy and the other to lower energy by the same amount as the result of the off-diagonal matrix element. We model the absorption profile by using Lorentzian functions, these profiles are then converted to transmission spectra (Figure 7(a)). The magnitude of this shift is then systematically varied, and the transmission curves with and without the introduced splitting are divided to obtain the division spectra. Finally, we normalize the division spectra based on the peak-to-valley value as shown in Figure 7(b).

**Figure 7:**
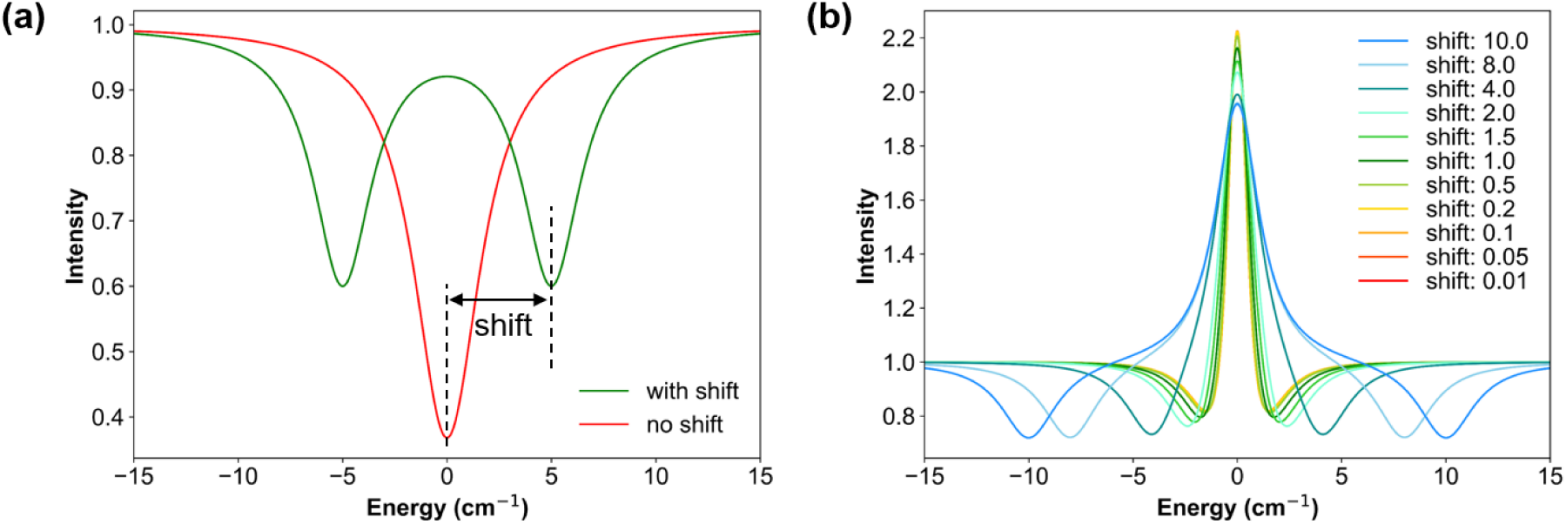
Mathematical model for division spectra. (a) The absorption profile (red) and splitting pattern with 5 cm^–1^ shift (green). fwhm = 3 cm^–1^. (b) Normalized division spectra with different shifts, varying from 0.01 to 10.0 cm^–1^.

When this shift approaches 0 cm^–1^, the two curves become increasingly identical and eventually the division converges to a straight line of value 1. In this regime, the normalization, based on the peak-to-valley value of the division spectrum, effectively highlights subtle features, such as the characteristic second-derivative structure in the field-division spectra. As illustrated in Figure 7(b), when the shift exceeds the line width, the separation between two minima in the normalized division spectra closely corresponds to twice the shift. In contrast, when the shift becomes smaller than the line width, the overall shape of the normalized spectrum remains almost unchanged, and the distance between the two minima asymptotically approaches the line width. This observation has a fundamental implication: when the spin–phonon coupling parameter is smaller than the experimental line width, there is a realistic risk that the apparent splitting inferred from the distance between the two minima in the “w”-shaped second derivative signal merely reflects the line width itself.

Thus, determining the magnitude of the spin–phonon coupling is not straightforward, especially when the coupling is smaller than the line width. Fortunately, the above mathematical exercise also suggests a method to estimate its magnitude, which is by focusing on the amplitude of the second derivative structure. Following this line of thought, we present simulated transmission and division spectra of the above one-phonon model by varying the spin–phonon coupling strength and phonon intensity shown in Figure 8 and Figures S3–S5. Here, by varying the spin–phonon coupling parameter *E* ^α^ over 1 order of magnitude, from 0.01 to 0.25 cm^–1^, the amplitude of the second-derivative structure varies tremendously. When the spin–phonon coupling becomes comparable to the spectral line width, the intensity borrowing effect becomes pronounced. In this regime, the resulting second derivative structure in the field-division spectra can exceed the amplitude of the original EPR signal, and a clear splitting of the phonon peak emerges in the transmission spectra. As the spin–phonon coupling is systematically reduced, the amplitude of the second derivative structure reduces correspondingly, eventually vanishing entirely when the coupling becomes too weak to induce noticeable mixing. For a fixed spin–phonon coupling, an increase in phonon intensity also leads to a larger second derivative structure in the field-division spectrum (Figure S5). This is also a direct consequence of enhanced intensity borrowing: stronger phonons more effectively amplify the spectral signatures of spin–phonon interactions. These observations highlight the sensitivity of field-division spectra to the magnitude of spin–phonon interactions and emphasize the challenges of detecting weak couplings in the presence of broad line widths.

**Figure 8:**
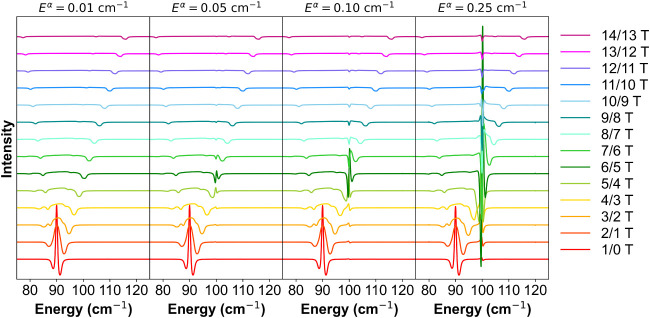
Field-division spectra of the one-phonon model. The spin–phonon coupling parameter *E* ^α^ is systematically varied from 0.01 cm^–1^ to 0.25 cm^–1^.

The above mathematical example clearly illustrates that it is not reliable to directly extract the spin–phonon coupling parameters from the distance between two minima in the characteristic “w”-shaped signal of the field-division spectra. The magnitude of this structure is determined not only by the spin–phonon coupling but also the intensity of the phonon. Therefore, the most robust approach involves first determining the relative intensities of the phonon and EPR transitions from the transmission data. With this information in hand, one can then simulate the amplitude of the second derivative structure in the field division spectra to most accurately extract the spin–phonon coupling parameters.

In addition to the *E* ^α^ parameter, the $g_{x}^{\alpha }$ and $g_{y}^{\alpha }$ are responsible for field-dependent off-diagonal elements that induce coupling between levels with Δ*M*_*S*_ = ±1. Conversely, the $g_{z}^{\alpha }$ and *D* ^α^ serve as diagonal matrix elements and are less effective in mediating transitions between different *M*_*S*_ levels. Our analysis indicates that for this *S* = 3/2 system, the most significant spin–phonon coupling parameter is *E* ^α^, which results in the most prominent interference patterns within the spectra. We refer to the Supporting Information for a comprehensive discussion of the contribution of the other spin–phonon coupling parameters.

### Simulation of Complex **1**

Now we turn to the simulation of the experimental spectra of complex **1**. Based on the above investigations, a strong correlation is expected between the spin–phonon coupling strength and the relative intensity of spin-flip transitions and phonons. To quantitatively simulate the field-division spectra incorporating spin–phonon coupling, the first step involves determining the relative intensity of spin-flip and phonon absorptions. We thus begin by focusing on the observed phonon signatures in the raw transmission data. We attribute these features to intrinsic phonon absorptions from the sample. We simulate the relative intensities of the spin-flip and phonon bands, as shown in Figure 9. A Lorentzian line shape was used with a fixed line width of 1.6 cm^–1^ for both spin-flip and phonon transitions.

**Figure 9:**
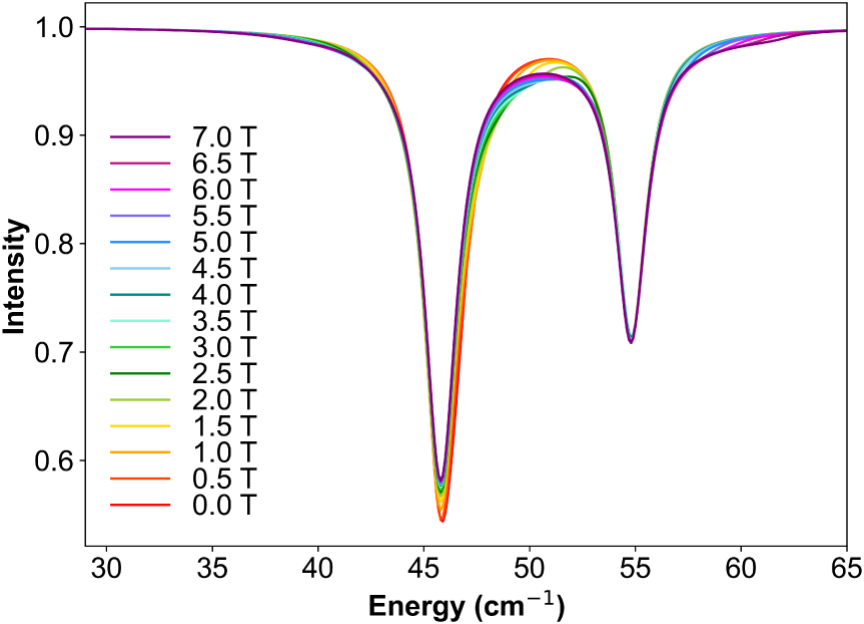
Simulation of powder transmission spectra for **1** with two phonons, *T* = 5 K, Lorentzian line width = 1.6 cm^–1^, Voigt mode, unpolarized light.

The second step involves optimizing the spin Hamiltonian parameters to reproduce the spectral branches upon elevating the field, regardless of spin–phonon coupling structures. Building upon insights from previous THz-EPR investigations of **1**,^55^ we adopted a previously determined set of spin Hamiltonian parameters, with slight refinements to better match our current data. The resulting parameters are as follows: *D* = −23.0 cm^–1^, *E* = −1.9 cm^–1^, *g*_*xx*_ = *g*_*yy*_ = 2.11, *g_zz_* = 2.40.

The final step involves tuning the spin–phonon coupling strength to best reproduce the second-derivative spectral features. While the phonon at 45.8 cm^–1^ may exhibit weak spin–phonon coupling, the observed second derivative structure is comparable to the noise level, making definitive assignment ambiguous. Therefore, the spin–phonon coupling parameter *E* ^α^ for this mode was constrained to zero. In contrast, the 54.8 cm^–1^ phonon displays a pronounced second-derivative-like structure upon increasing the external field from 0 to 4 T, which then gradually diminishes at higher fields. This behavior suggests a dominant contribution from the field-independent term *E* ^α^, rather than from field-dependent term $g_{i}^{\alpha }$ . By adjusting the coupling strength, we successfully simulate the field-division spectra (as shown in Figure 10) and estimate the spin–phonon coupling parameter *E* ^α^ to be approximately 0.16 cm^–1^. This parameter is indeed significantly smaller than the intrinsic line width, yet sufficient to reproduce the amplitude of the observed second-derivative structure.

**Figure 10:**
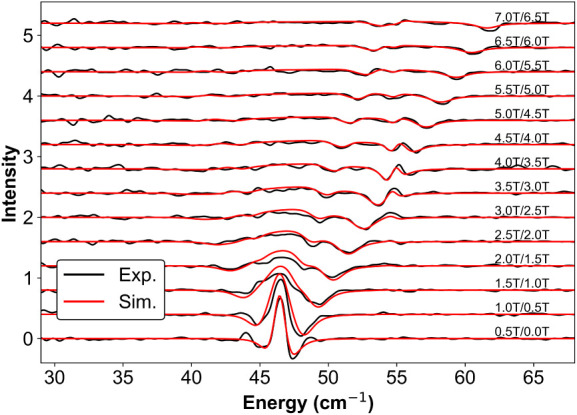
Simulated division spectra of **1**, *T* = 5 K, Voigt mode, unpolarized light, with *E* ^α^ = 0.16 cm^–1^, Lorentzian line width = 1.6 cm^–1^.

### Theoretical Aspects of Spin Phonon Coupling

Our previous simulations provided an estimate of the spin–phonon coupling parameter *E* ^α^ for **1**. A natural extension of this work is to examine whether *ab initio* calculations can yield further insights that not only complement our simulations but also clarify which vibrational modes play a dominant role in mediating spin–phonon couplings.

In our framework, spin–phonon coupling is considered as the perturbation of the electronic structure by vibrational motions that in turn modulate spin-dependent properties. In Werner-type 3d transition metal systems, the spin-Hamiltonian parameters are predominantly determined by the relative energies and occupations of the five 3d orbitals. Consequently, the most critical effect of vibrational motion arises from the modulation of the 3d-orbital energy levels. Ligand-field theory offers an appropriate conceptual framework for understanding this process.^85^ Our analysis therefore begins by evaluating how distinct vibrational modes affect the relative 3d-orbital energy levels in a tetrahedral coordinated system.

### Vibrational Modes of a Tetrahedral Model System

We first consider the tetrahedral complex [CoCl_4_]^2–^ as a model system. It exhibits four classes of vibrational modes: symmetric bending, asymmetric bending, symmetric stretching, and asymmetric stretching (Figure 11). Among these, two “distinct” symmetric bending modes can be distinguished. The first reduces the symmetry from *T*_d_ to *S*_4_ and corresponds to an in-plane Cl–Co–Cl angular deformation, hereafter referred to as the scissoring mode. The second lowers the symmetry to D_2_ and involves angular distortion between two perpendicular Cl–Co–Cl planes, which we denote as the twisting mode. Although these two modes belong to the same irreducible representation in *T*_d_ symmetry, realistic ligand environments usually remove this degeneracy.

**Figure 11:**
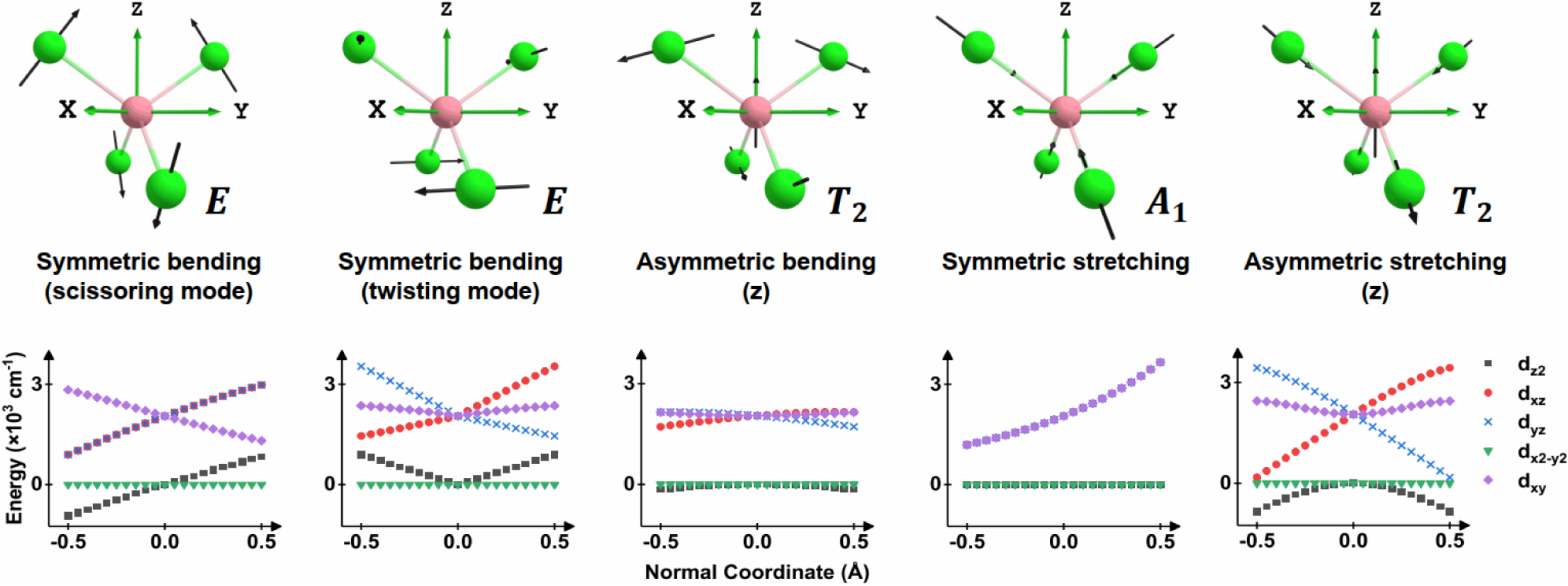
Fundamental vibrational modes of the first coordination sphere and their modulation to the AILFT d-orbital energy levels. The d_*x*2–_ *_y_*_2_ energy level is used as the reference. The d*_xz_*_/*yz*_ energy levels mix for asymmetric bending and stretching and become in-phase and out-of-phase linear combinations. The irreducible representations under *T*_d_ symmetry are indicated alongside each normal mode.

Ligand field theory provides a useful qualitative framework for assessing how such vibrational modes perturb the energy levels of the 3d orbitals. Vibrational motions that enhance either angular or radial overlap between ligand orbitals and a given metal d orbital result in destabilization of that d orbital energy. In (near-)­tetrahedral coordination, the d_*z*2_ and d_*x*2–_ *_y_*_2_ orbitals remain essentially nonbonding and are therefore relatively weakly affected by distortions. By contrast, the t_2_ set (d_*xy*_, d_*xz*_, d_*yz*_) is more significantly modulated.

Figure 11 summarizes the modulation of the 3d energy levels of these vibrational modes in [CoCl_4_]^2–^. For example, in equilibrium the ∠Cl–Co–Cl angle is 109.5° and the positive and negative lobe of d_*xz*_ is oriented 90° toward each other. Therefore, the scissoring mode that reduces or enlarges this bond angle would enhance or decrease the angular overlap with the d_*xz*_ orbital, thus modulating its energy. Due to the symmetry, the scissoring mode preserves the degeneracy of d_*xz*_/d_*yz*_ orbitals and destabilizes them both while at the same time it stabilizes the d_*xy*_ orbital. Conversely, the twisting mode primarily lifts the degeneracy between the d_*xz*_ and d_*yz*_ orbitals. The symmetric stretching mode alters the Co–Cl bond distances and thus modulates the radial overlap between the ligand orbitals and the t_2_ set (d_*xy*_, d_*xz*_, d_*yz*_). Hence, this mode does not alter orbital degeneracies but modulates the overall ligand-field strength. The asymmetric bending and asymmetric stretching modes act in a similar fashion: they displace the metal ion along one direction (*x*, *y*, or *z*), while the ligands move in the opposite direction. This displacement selectively affects the two orbitals that have the axis of motion in their nodal notation (e.g., motion along *x* predominantly splits d_*xy*_ and d_*xz*_).

Together, these results establish a direct correlation between specific vibrational modes and the characteristic changes they induce to the d-orbital energy levels. In more structurally complex tetrahedral molecular systems of lower symmetry, such as complex **1**, the situation becomes less straightforward. Nevertheless, the low-frequency vibrational modes can still be decomposed into the four fundamental classes identified in the [CoCl_4_]^2–^ model: symmetric and asymmetric bending and stretching. In practice, however, these modes appear as linear combinations mixed with motions of the extended ligand scaffold. This coupling to the broader ligand environment dilutes the contribution of the pure first-coordination sphere motions, generally leading to weaker modulation of the five d-orbital energy levels.

### Theoretical Assessment of the Spin-Hamiltonian Parameters of Complex **1**

Complex **1** features an elongated tetrahedral coordination geometry, with an optimized ∠N–Co–O angle of 96.7°. The corresponding ligand field splitting, obtained from NEVPT2-AILFT, is presented in Figure 12. As expected for a tetrahedral high-spin d^7^ configuration, the ground state is dominated by the configuration of (d_*z*2_)^2^(d_*x*2–_ *_y_*_2_)^2^(d_*xy*_)^1^(d_*xz*_)^1^(d_*yz*_)^1^. The compressed ∠N–Co–O angle imposed by the ligand framework destabilizes the d_*xz*_ and d_*yz*_ orbitals while stabilizing the d_*xy*_ orbital, resulting in a near-axial system with moderate splitting between d*_xz_*_/*yz*_ and d_*xy*_.

**Figure 12:**
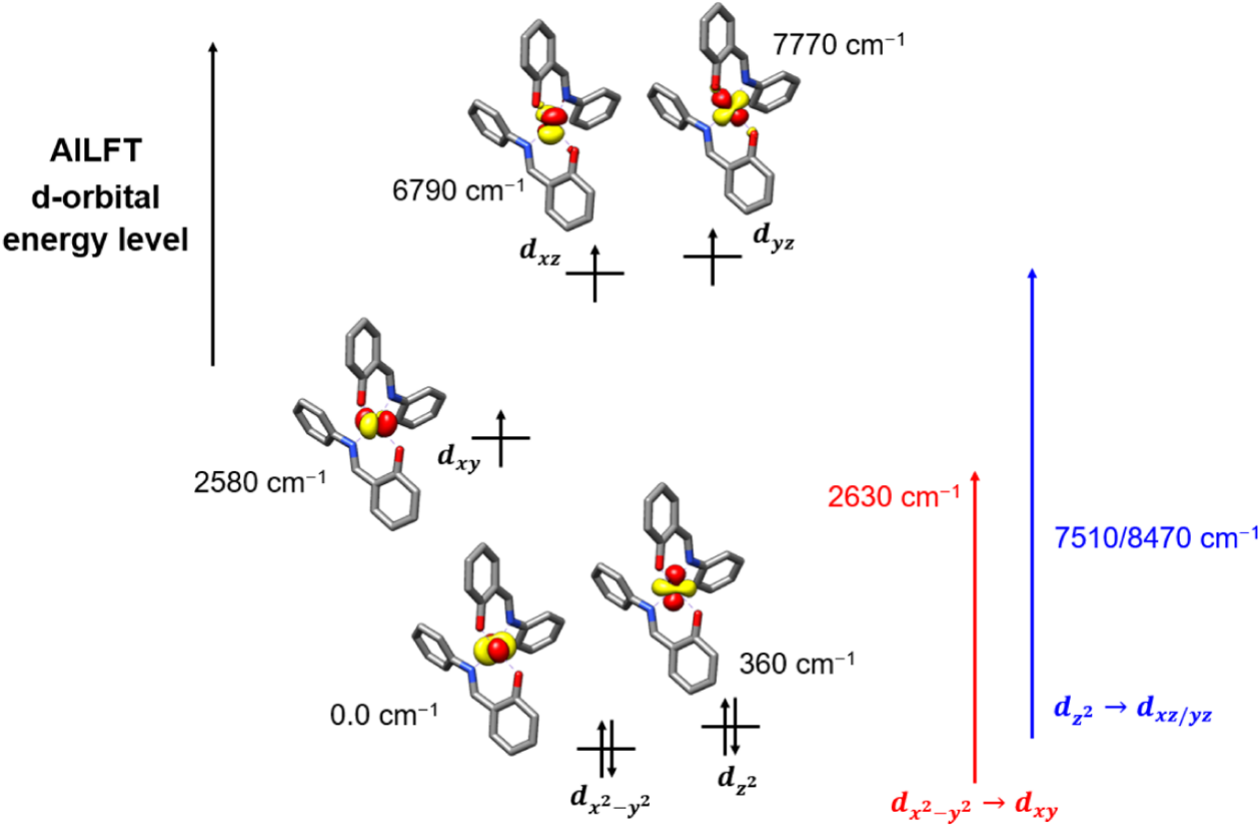
AILFT results of complex **1** and the three low-lying excited states.

In 3d transition metal systems, the ZFS and *g*-tensor are primarily governed by spin–orbit coupling (SOC) within the 3d manifold. Contributions to the ZFS arise from SOC within the same spin multiplicity (Δ*S* = 0) as well as those involving Δ*S* = ± 1; however, the latter contributions are negligible in the case of a high-spin d^7^ system. A detailed analysis of all excited states has been provided in the Supporting Information, and for complex **1**, three low-lying excited states dominate the SOC pattern (Figure 12), each of which can be effectively described as a single-electron excitation.

The lowest excitation involves promotion of an electron from the d_*x*2–_ *_y_*_2_ orbital to the d_*xy*_ orbital, leading to the dominant configuration of (d_*z*2_)^2^(d_*x*2–_ *_y_*_2_)^1^(d_*xy*_)^2^(d_*xz*_)^1^(d_*yz*_)^1^. SOC between this excited state and the ground state generates substantial unquenched orbital angular momentum along the molecular *z*-axis, producing a negative ZFS (*D* < 0) and a significant positive *g*-shift along *z*. The next two excitations involve promoting an electron from the d_*z*2_ orbital to the d*_xz_*_/*yz*_ pair, thereby introducing orbital angular momentum along the *x* and *y* axes. However, these contributions are weaker because second order mixing by SOC scales inversely with excitation energy. As a result, they give rise to smaller positive *g*-shifts along *x* and *y* axes and, owing to the near degeneracy of d_*xz*_ and d_*yz*_, induce only minimal rhombicity (*E*/*D* ≈ 0). The *ab initio* calculations predict spin Hamiltonian parameters of *D* = −28.0 cm^–1^, |*E*| = 0.6 cm^–1^, *g_xx_* = 2.13, *g_yy_* = 2.15, *g_zz_* = 2.47, in agreement with the above electronic structure analysis and shows excellent agreement with experimental values.

### Vibrational Mode Responsible for Large Spin–Phonon Coupling

The lowest excitation (d_*x*2–_ *_y_*_2_ → d_*xy*_) gives rise to a negative *D* value, while the d_*z*2_ → d*_xz_*_/*yz*_ excitations govern the rhombicity. Thus, vibrational modes that modulate these excitation energies are expected to dominate the spin–phonon coupling. The frequency calculation of **1** shows that only first-coordination sphere bending modes are observed in the low-frequency region (< 200 cm^–1^). Stretching modes are found at higher frequency. Table S5 summarizes their calculated spin–phonon coupling parameters. In this near axial case, there’s almost no rotation of the principal axes of *D*-tensor for these vibrational modes, supporting our earlier approximation.

The symmetric bending (twisting) mode stands out with a large spin–phonon coupling parameter *E* ^α^ of 0.28 cm^–1^ and a vibrational frequency of 60 cm^–1^, close to the experimentally observed phonon. This mode primarily perturbs the d_*xz*_ and d_*yz*_ energy levels as shown in Figure 13, directly modulating the rhombicity parameter *E*. Such a symmetric bending mode is IR-forbidden in ideal axial symmetry, but the difference between nitrogen and oxygen coordination renders it weakly active with the electric transition dipole moment situated in the *xy* plane, so that it becomes comparable in intensity to the EPR transition.

**Figure 13:**
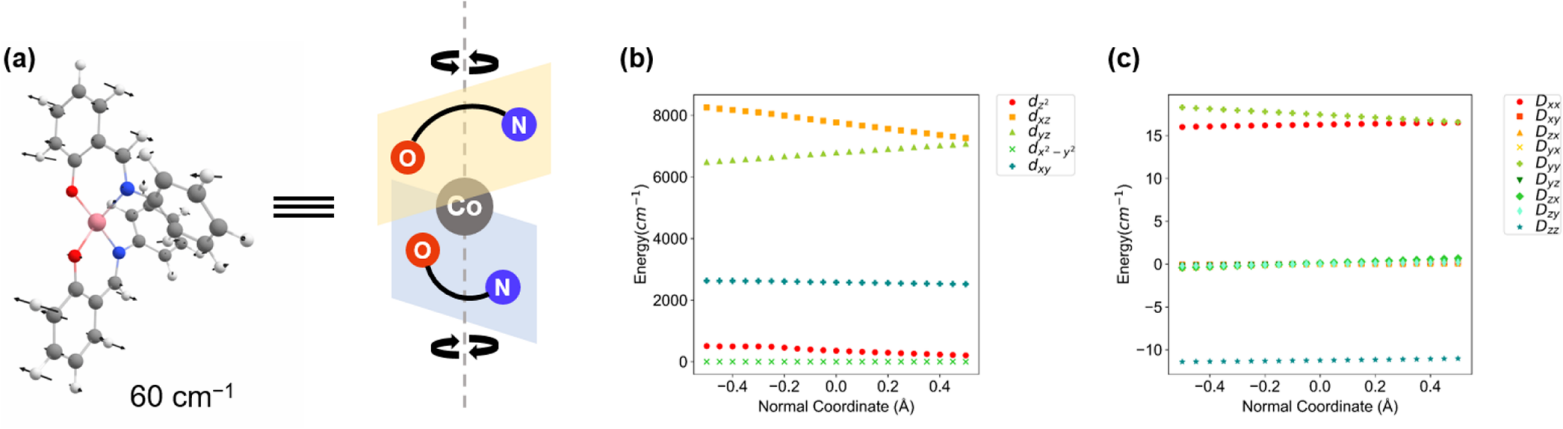
(a) The symmetric bending (twisting) mode at 60 cm^–1^. Modulation of (b) d-energy levels and (c) *D*-tensor of the lowest symmetric bending (twisting) mode.

Although precise calculation of low-frequency vibrations remains challenging, our analysis allows a confident assignment of the 55 cm^–1^ phonon observed experimentally to the symmetric bending (twisting) mode of the two N–Co–O fragments (cf. Figure 13). The calculated spin–phonon coupling parameter *E* ^α^ of 0.28 cm^–1^ is reasonably close to the simulated *E* ^α^ of 0.16 cm^–1^, both of which are significantly smaller than the experimental line width. This consistency between experiment, simulation, and calculation provides strong support for our newly developed protocol as a reliable approach for extracting spin–phonon coupling information from magnetic IR/THz spectra.

## Conclusions

In this contribution we have established a formalism based on an extended effective spin Hamiltonian to simulate THz EPR spectra including amplitudes of the observed signals and including spin–phonon coupling. Using this formalism, we were able for the first time to accurately simulate the observed field dependence in the THz EPR spectra of an exemplary molecule, **1**. We have extracted an accurate value the spin–phonon coupling parameter *E* ^α^ = 0.16 cm^–1^. This number is significantly smaller than the Lorentzian line width observed in the spectrum (1.6 cm^–1^). It is of crucial importance in this respect that the distance of minimum approach in the avoided crossings generally corresponds to the line width rather than twice the coupling element when the coupling element is smaller than the line width. We therefore believe that simulation of the field dependence and the amplitudes together is critical to obtain accurate spin phonon coupling constants. Parallel quantum chemical calculations and subsequent analysis yield results that are consistent with the simulations. As such, we believe that the presented formalism is generally useful for the field and the example provided here serves as a solid proof-of-principle of the validity of our simulation procedure. Lastly, it also establishes THz EPR spectroscopy as a unique technique by which it is possible to obtain direct information about spin phonon coupling by experiment and concomitant analysis. While THz EPR spectroscopy, as a one-photon absorption spectroscopy, is not sensitive to follow up energy transfer and relaxation processes relevant for quantum computing, it does serve as an experimental means to measure coupling strengths that are otherwise only accessible from quantum chemical calculations. Parameter extraction from experiment of the kind we have demonstrated here will hopefully serve as an invaluable bridge between theory and experiment as the theoretical methods to predict spin–phonon coupling parameters become increasingly available.

## Supplementary Material



## Data Availability

ORCA input files and scripts for processing theoretical data are available at the Edmond repository at 10.17617/3.AIUEMB.

## Notes

Detailed definition of spin–phonon coupling parameters, explicit expression of the matrix elements of the total Hamiltonian, unit conversion, details about the filtration procedure, additional simulations, calculated parameters by CASSCF/NEVPT2 calculations, and Cartesian coordinates of the employed models (PDF)

## References

[1] Atzori M., Sessoli R. (2019). The Second Quantum Revolution: Role and Challenges
of Molecular Chemistry. J. Am. Chem. Soc..

[2] Yu C. J., von Kugelgen S., Laorenza D. W., Freedman D. E. (2021). A Molecular Approach
to Quantum Sensing. ACS Cent. Sci..

[3] Wasielewski M. R., Forbes M. D. E., Frank N. L., Kowalski K., Scholes G. D., Yuen-Zhou J., Baldo M. A., Freedman D. E., Goldsmith R. H., Goodson T. (2020). Exploiting chemistry
and molecular systems for quantum information science. Nat. Rev. Chem..

[4] Atzori M., Tesi L., Morra E., Chiesa M., Sorace L., Sessoli R. (2016). Room-Temperature Quantum Coherence
and Rabi Oscillations
in Vanadyl Phthalocyanine: Toward Multifunctional Molecular Spin Qubits. J. Am. Chem. Soc..

[5] Lunghi A., Sanvito S. (2020). The Limit of Spin Lifetime
in Solid-State Electronic
Spins. J. Phys. Chem. Lett..

[6] Mirzoyan R., Kazmierczak N. P., Hadt R. G. (2021). Deconvolving Contributions to Decoherence
in Molecular Electron Spin Qubits: A Dynamic Ligand Field Approach. Chemistry.

[7] Escalera-Moreno L., Baldovi J. J., Gaita-Arino A., Coronado E. (2018). Spin states, vibrations
and spin relaxation in molecular nanomagnets and spin qubits: a critical
perspective. Chem. Sci..

[8] Sessoli R., Gatteschi D., Caneschi A., Novak M. A. (1993). Magnetic bistability
in a metal-ion cluster. Nature.

[9] Zadrozny J.
M., Niklas J., Poluektov O. G., Freedman D. E. (2015). Millisecond Coherence
Time in a Tunable Molecular Electronic Spin Qubit. ACS Cent. Sci..

[10] Donati F., Rusponi S., Stepanow S., Wäckerlin C., Singha A., Persichetti L., Baltic R., Diller K., Patthey F., Fernandes E., Dreiser J., Šljivančanin Š., Kummer K., Nistor C., Gambardella P., Brune H. (2016). Magnetic remanence
in single atoms. Science.

[11] Gupta S. K., Rajeshkumar T., Rajaraman G., Murugavel R. (2016). An air-stable
Dy­(iii) single-ion magnet with high anisotropy barrier and blocking
temperature. Chem. Sci..

[12] Chen Y., Ma F., Chen X., Dong B., Wang K., Jiang S., Wang C., Chen X., Qi D., Sun H., Wang B., Gao S., Jiang J. (2017). A New Bis­(phthalocyaninato)
Terbium Single-Ion Magnet with an Overall Excellent Magnetic Performance. Inorg. Chem..

[13] Goodwin C. A. P., Ortu F., Reta D., Chilton N. F., Mills D. P. (2017). Molecular
magnetic hysteresis at 60 K in dysprosocenium. Nature.

[14] Guo F. S., Day B. M., Chen Y. C., Tong M. L., Mansikkamaki A., Layfield R. A. (2017). A Dysprosium Metallocene Single-Molecule Magnet Functioning
at the Axial Limit. Angew. Chem., Int. Ed..

[15] Sessoli R. (2017). Magnetic molecules
back in the race. Nature.

[16] Fataftah M. S., Krzyaniak M. D., Vlaisavljevich B., Wasielewski M. R., Zadrozny J. M., Freedman D. E. (2019). Metal-ligand
covalency enables room
temperature molecular qubit candidates. Chem.
Sci..

[17] Zhu Z., Ying X., Zhao C., Zhang Y.-Q., Tang J. (2022). A new breakthrough
in low-coordinate Dy­(III) single-molecule magnets. Inorg. Chem. Front..

[18] Gould C. A., McClain K. R., Reta D., Kragskow J. G. C., Marchiori D. A., Lachman E., Choi E.-S., Analytis J. G., Britt R. D., Chilton N. F., Harvey B. G., Long J. R. (2022). Ultrahard magnetism
from mixed-valence dilanthanide complexes with metal-metal bonding. Science.

[19] Juráková J., Dubnická
Midlíková J., Hrubý J., Kliuikov A., Santana V. T., Pavlik J., Moncoĺ J., Čižmár E., Orlita M., Mohelský I., Neugebauer P., Gentili D., Cavallini M., Šalitroš I. (2022). Pentacoordinate cobalt­(ii) single
ion magnets with pendant alkyl chains: shall we go for chloride or
bromide?. Inorg. Chem. Front..

[20] Zhang P., Nabi R., Staab J. K., Chilton N. F., Demir S. (2023). Taming Super-Reduced
Bi(2)­(3-) Radicals with Rare Earth Cations. J. Am. Chem. Soc..

[21] Bernbeck M. G., Orlova A. P., Hilgar J. D., Gembicky M., Ozerov M., Rinehart J. D. (2024). Dipolar Coupling
as a Mechanism for Fine Control of
Magnetic States in ErCOT-Alkyl Molecular Magnets. J. Am. Chem. Soc..

[22] Benner F., Jena R., Odom A. L., Demir S. (2025). Magnetic Hysteresis
in a Dysprosium Bis­(amide) Complex. J. Am. Chem.
Soc..

[23] Bloch F. (1946). Nuclear Induction. Phys. Rev..

[24] Standley, K. J. ; Vaughan, R. A. Electron Spin Relaxation Phenomena in Solids; Springer US, 1969.

[25] Van
Vleck J. H. (1940). Paramagnetic Relaxation Times for Titanium and Chrome
Alum. Phys. Rev..

[26] Orbach R. (1961). Spin-lattice
relaxation in rare-earth salts. Proc. R. Soc.
London A.

[27] Lunghi A. (2023). Spin-Phonon
Relaxation in Magnetic Molecules: Theory, Predictions and Insights. Comput. Model. Mole. Nanomag..

[28] Kragskow J. G. C., Mattioni A., Staab J. K., Reta D., Skelton J. M., Chilton N. F. (2023). Spin-phonon coupling
and magnetic relaxation in single-molecule
magnets. Chem. Soc. Rev..

[29] Neue G., Bai S., Taylor R. E., Beckmann P. A., Vega A. J., Dybowski C. (2009). ^119^Sn spin-lattice
relaxation in alpha-SnF_2_. Phys. Rev.
B.

[30] Ward R., Bowman A., Sozudogru E., El-Mkami H., Owen-Hughes T., Norman D. G. (2010). EPR distance measurements in deuterated proteins. J. Magn. Reson..

[31] Escalera-Moreno L., Suaud N., Gaita-Arino A., Coronado E. (2017). Determining Key Local
Vibrations in the Relaxation of Molecular Spin Qubits and Single-Molecule
Magnets. J. Phys. Chem. Lett..

[32] Lunghi A., Sanvito S. (2019). How do phonons relax
molecular spins?. Sci. Adv..

[33] Santanni F., Albino A., Atzori M., Ranieri D., Salvadori E., Chiesa M., Lunghi A., Bencini A., Sorace L., Totti F., Sessoli R. (2021). Probing Vibrational
Symmetry Effects
and Nuclear Spin Economy Principles in Molecular Spin Qubits. Inorg. Chem..

[34] Kazmierczak N. P., Mirzoyan R., Hadt R. G. (2021). The Impact of Ligand
Field Symmetry
on Molecular Qubit Coherence. J. Am. Chem. Soc..

[35] Kazmierczak N. P., Hadt R. G. (2022). Illuminating Ligand Field Contributions to Molecular
Qubit Spin Relaxation via T(1) Anisotropy. J.
Am. Chem. Soc..

[36] Garlatti E., Albino A., Chicco S., Nguyen V. H. A., Santanni F., Paolasini L., Mazzoli C., Caciuffo R., Totti F., Santini P. (2023). The critical role of ultra-low-energy vibrations in
the relaxation dynamics of molecular qubits. Nat. Commun..

[37] Kazmierczak N. P., Oyala P. H., Hadt R. G. (2024). Spectroscopic
Signatures of Phonon
Character in Molecular Electron Spin Relaxation. ACS Cent. Sci..

[38] Shushkov P. (2024). A novel non-adiabatic
spin relaxation mechanism in molecular qubits. J. Chem. Phys..

[39] Kazmierczak N. P., Lopez N. E., Luedecke K. M., Hadt R. G. (2024). Determining the
key vibrations for spin relaxation in ruffled Cu­(ii) porphyrins via
resonance Raman spectroscopy. Chem. Sci..

[40] Mariano L.
A., Nguyen V. H. A., Petersen J. B., Björnsson M., Bendix J., Eaton G. R., Eaton S. S., Lunghi A. (2025). The role of
electronic excited states in the spin-lattice relaxation of spin-1/2
molecules. Sci. Adv..

[41] Kazmierczak N. P., Xia K. T., Sutcliffe E., Aalto J. P., Hadt R. G. (2025). A Spectrochemical
Series for Electron Spin Relaxation. J. Am.
Chem. Soc..

[42] Holldack K., Schnegg A. (2016). THz Electron Paramagnetic Resonance/THz Spectroscopy
at BESSY II. J. Large Scale Res. Faci..

[43] Nehrkorn J., Holldack K., Bittl R., Schnegg A. (2017). Recent progress in
synchrotron-based frequency-domain Fourier-transform THz-EPR. J. Magn. Reson..

[44] Malinová N., Juráková J., Brachňaková B., Midlíková J. D., Čižmár E., Santana V. T., Herchel R., Orlita M., Mohelský I., Moncol J., Neugebauer P., Šalitroš I. (2023). Magnetization
Slow Dynamics in Mononuclear Co­(II) Field-Induced Single-Molecule
Magnet. Cryst. Growth Des..

[45] Kuhne P., Herzinger C. M., Schubert M., Woollam J. A., Hofmann T. (2014). Invited article:
An integrated mid-infrared, far-infrared, and terahertz optical Hall
effect instrument. Rev. Sci. Instrum..

[46] Wiegers S. A. J., Christianen P. C. M., Engelkamp H., den Ouden A., Perenboom J. A. A. J., Zeitler U., Maan J. C. (2010). The High
Field Magnet Laboratory at Radboud University Nijmegen. J. Low Temp. Phys..

[47] Mihály L., Talbayev D., Kiss L. F., Zhou J., Fehér T., Jánossy A. (2004). Field-frequency
mapping of the electron spin resonance
in the paramagnetic and antiferromagnetic states of LaMnO_3_. Phys. Rev. B.

[48] Takehana K., Oshikiri M., Kido G., Takazawa A., Sato M., Nagasaka K., Hase M., Uchinokura K. (1996). Far-infrared
spectroscopy in high magnetic fields. Phys.
B.

[49] Padilla W. J., Li Z. Q., Burch K. S., Lee Y. S., Mikolaitis K. J., Basov D. N. (2004). Broadband multi-interferometer spectroscopy
in high
magnetic fields: From THz to visible. Rev. Sci.
Instrum..

[50] Kragskow J. G. C., Marbey J., Buch C. D., Nehrkorn J., Ozerov M., Piligkos S., Hill S., Chilton N. F. (2022). Analysis of vibronic
coupling in a 4f molecular magnet with FIRMS. Nat. Commun..

[51] Moseley D. H., Stavretis S. E., Thirunavukkuarasu K., Ozerov M., Cheng Y., Daemen L. L., Ludwig J., Lu Z., Smirnov D., Brown C. M. (2018). Spin-phonon couplings in transition metal complexes
with slow magnetic relaxation. Nat. Commun..

[52] Wan Y., Cheng X., Li Y., Wang Y., Du Y., Zhao Y., Peng B., Dai L., Kan E. (2021). Manipulating
the Raman scattering rotation via magnetic field in an MoS_2_ monolayer. RSC Adv..

[53] Kong X., Ganesh P., Liang L. (2024). First-principles
study of the magneto-Raman
effect in van der Waals layered magnets. Npj
2D Mater. Appl..

[54] McCreary A., Mai T. T., Utermohlen F. G., Simpson J. R., Garrity K. F., Feng X., Shcherbakov D., Zhu Y., Hu J., Weber D. (2020). Distinct magneto-Raman
signatures of spin-flip phase
transitions in CrI(3). Nat. Commun..

[55] Pohle M. H., Lohmiller T., Bohme M., Rams M., Ziegenbalg S., Gorls H., Schnegg A., Plass W. (2024). THz-EPR-based
Magneto-Structural
Correlations for Cobalt­(II) Single-Ion Magnets With Bis-Chelate Coordination. Chem. - Eur. J..

[56] Bone A. N., Widener C. N., Moseley D. H., Liu Z., Lu Z., Cheng Y., Daemen L. L., Ozerov M., Telser J., Thirunavukkuarasu K., Smirnov D., Greer S. M., Hill S., Krzystek J., Holldack K., Aliabadi A., Schnegg A., Dunbar K. R., Xue Z. L. (2021). Applying Unconventional Spectroscopies
to the Single-Molecule Magnets, Co­(PPh(3))(2) X(2) (X = Cl, Br, I):
Unveiling Magnetic Transitions and Spin-Phonon Coupling. Chem. - Eur. J..

[57] Mondal S., Netz J., Hunger D., Suhr S., Sarkar B., van Slageren J., Kohn A., Lunghi A. (2025). The Spin-Phonon Relaxation
Mechanism of Single-Molecule Magnets in the Presence of Strong Exchange
Coupling. ACS Cent. Sci..

[58] Crassee I., Levallois J., Walter A. L., Ostler M., Bostwick A., Rotenberg E., Seyller T., van der Marel D., Kuzmenko A. B. (2011). Giant Faraday rotation
in single- and multilayer graphene. Nat. Phys..

[59] Earle K. A., Tipikin D. S., Freed J. H. (1996). Far-infrared
electron-paramagnetic-resonance
spectrometer utilizing a quasioptical reflection bridge. Rev. Sci. Instrum..

[60] Joyce R. R., Richards P. L. (1969). Far-Infrared Spectra
of Al2O3 Doped with Ti, V, and
Cr. Phys. Rev..

[61] Champion P. M., Sievers A. J. (1980). Far infrared magnetic
resonance of deoxyhemoglobin
and deoxymyoglobin. J. Chem. Phys..

[62] Richards P.
L., Caughey W. S., Eberspaecher H., Feher G., Malley M. (1967). Determination
of the Zero-Field Splitting of Fe3+ in Several Hemin Compounds. J. Chem. Phys..

[63] Hunger D., Netz J., Suhr S., Thirunavukkuarasu K., Engelkamp H., Fak B., Albold U., Beerhues J., Frey W., Hartenbach I. (2025). Electronic structure
of mononuclear and radical-bridged dinuclear cobalt­(II) single-molecule
magnets. Nat. Commun..

[64] Neese F. (2025). Software Update:
The ORCA Program System—Version 6.0. WIREs. Comput. Mol. Sci..

[65] Staroverov V. N., Scuseria G. E., Tao J., Perdew J. P. (2003). Comparative assessment
of a new nonempirical density functional: Molecules and hydrogen-bonded
complexes. J. Chem. Phys..

[66] Weigend F., Ahlrichs R. (2005). Balanced basis sets
of split valence, triple zeta valence
and quadruple zeta valence quality for H to Rn: Design and assessment
of accuracy. Phys. Chem. Chem. Phys..

[67] Grimme S., Ehrlich S., Goerigk L. (2011). Effect of
the damping function in
dispersion corrected density functional theory. J. Comput. Chem..

[68] Grimme S., Antony J., Ehrlich S., Krieg H. (2010). A consistent and accurate
ab initio parametrization of density functional dispersion correction
(DFT-D) for the 94 elements H-Pu. J. Chem. Phys..

[69] Izsák R., Neese F., Klopper W. (2013). Robust fitting techniques in the
chain of spheres approximation to the Fock exchange: The role of the
complementary space. J. Chem. Phys..

[70] Izsák R., Neese F. (2011). An overlap fitted chain of spheres exchange method. J. Chem. Phys..

[71] Neese F., Wennmohs F., Hansen A., Becker U. (2009). Efficient, approximate
and parallel Hartree–Fock and hybrid DFT calculations. A ‘chain-of-spheres’
algorithm for the Hartree–Fock exchange. Chem. Phys..

[72] Stoychev G. L., Auer A. A., Neese F. (2017). Automatic
Generation of Auxiliary
Basis Sets. J. Chem. Theory. Comput..

[73] Malmqvist P.-Å., Roos B. O. (1989). The CASSCF state interaction method. Chem. Phys. Lett..

[74] Angeli C., Cimiraglia R., Malrieu J.-P. (2002). n-electron valence state perturbation
theory: A spinless formulation and an efficient implementation of
the strongly contracted and of the partially contracted variants. J. Chem. Phys..

[75] Angeli C., Cimiraglia R., Evangelisti S., Leininger T., Malrieu J. P. (2001). Introduction of
n-electron valence states for multireference
perturbation theory. J. Chem. Phys..

[76] Pollak P., Weigend F. (2017). Segmented Contracted
Error-Consistent Basis Sets of
Double- and Triple-zeta Valence Quality for One- and Two-Component
Relativistic All-Electron Calculations. J. Chem.
Theory. Comput..

[77] Franzke Y. J., Tress R., Pazdera T. M., Weigend F. (2019). Error-consistent segmented
contracted all-electron relativistic basis sets of double- and triple-zeta
quality for NMR shielding constants. Phys. Chem.
Chem. Phys..

[78] Kutzelnigg W., Liu W. (2005). Quasirelativistic theory equivalent to fully relativistic theory. J. Chem. Phys..

[79] Liu W., Peng D. (2009). Exact two-component
Hamiltonians revisited. J. Chem. Phys..

[80] Atanasov, M. ; Ganyushin, D. ; Sivalingam, K. ; Neese, F. A Modern First-Principles View on Ligand Field Theory Through the Eyes of Correlated Multireference Wavefunctions. In Molecular Electronic Structures of Transition Metal Complexes II, Mingos, D. M. P. ; Day, P. ; Dahl, J. P. , Eds.; Springer: Berlin Heidelberg: Berlin, Heidelberg, 2012; pp. 149–220.

[81] Lang L., Atanasov M., Neese F. (2020). Improvement
of Ab Initio Ligand Field
Theory by Means of Multistate Perturbation Theory. J. Phys. Chem. A.

[82] Nehrkorn J., Telser J., Holldack K., Stoll S., Schnegg A. (2015). Simulating
Frequency-Domain Electron Paramagnetic Resonance: Bridging the Gap
between Experiment and Magnetic Parameters for High-Spin Transition-Metal
Ion Complexes. J. Phys. Chem. B.

[83] Goldfarb, D. ; Stoll, S. EPR Spectroscopy: Fundamentals and Methods. Wiley: 2018.

[84] Hagen, W. R. Biomolecular EPR Spectroscopy; CRC Press, 2008.

[85] Atanasov M., Aravena D., Suturina E., Bill E., Maganas D., Neese F. (2015). First principles approach to the
electronic structure, magnetic anisotropy
and spin relaxation in mononuclear 3d-transition metal single molecule
magnets. Coord. Chem. Rev..

