# Supplementary material for: Bridging Vibrations and Spins: Mode-Resolved Spin–Phonon Coupling Revealed through THz EPR/Magnetic IR Simulation
Source: J Phys Chem A. 2026 Feb 10;130(7):1486–500. doi: 10.1021/acs.jpca.5c07944 (PMC12927012; doi:10.1021/acs.jpca.5c07944)
Supplement: Supplementary file 1 [file jp5c07944_si_001.pdf]

# Bridging Vibrations and Spins: Mode-Resolved Spin-Phonon Coupling Revealed through THz EPR/Magnetic IR Simulation

Haowei Chen[1], Maurice van Gastel[1], Alexander Schnegg[2], Frank Neese[1,*]

[1] Department of Molecular Theory and Spectroscopy, Max-Planck-Institut für Kohlenforschung, Kaiser Wilhelmplatz 1, 45470 Mülheim an der Ruhr, Germany

[2] EPR Research group, Max-Planck-Institut für Chemische Energiekonversion, Stiftstrasse 34-36, 45470, Mülheim an der Ruhr, Germany

# Supporting Information

## Contents

Detailed definition of spin-phonon coupling parameters ........ 3

Explicit expression of the matrix elements of $\hat{H}_{tot}$ in $|\boldsymbol{v},SM_S\rangle$ basis ........ 4

Unit conversion of spin-phonon coupling parameters ........ 7

Raw Experimental Data ........ 8

High-frequency filtration of raw transmission data ........ 9

Additional simulations ........ 11

  Varying spin-phonon coupling strength and phonon transition intensity ........ 11

  Orientation of the electric transition dipole moment ........ 12

  The contribution of $g^{\alpha}_{x/y}$ ........ 13

  The contribution of $D^{\alpha}$ and $g^{\alpha}_{z}$ ........ 13

  Sign of zero-field splitting ........ 15

Theoretical Model of $[CoCl_4]^{2-}$ ........ 18

Theoretical Model of $[Co(ndh)_2]^{2-}$ ........ 21

Theoretical Model of complex **1** ........ 26

Molecular Coordinate ........ 30

## Detailed definition of spin-phonon coupling parameters

The spin-phonon coupling terms serve as off-diagonal-block matrix elements in the Hamiltonian matrix. They not only contain the derivatives of $g_i$, $D$, and $E$ over a specific normal mode, but also includes the integral over that normal mode. For example, the matrix element related to $\left(\frac{\partial g_i}{\partial Q_\alpha}\right)$ under $|\vec{v}, SM_S\rangle$ basis (consider only normal mode $\alpha$) gives the following integral:

$$\left\langle n_\alpha, SM_S \middle| \mu_B B_0 l_i \left(\frac{\partial g_i}{\partial Q_\alpha}\right) \hat{Q}_\alpha \hat{S}_i \middle| n'_\alpha, SM'_S \right\rangle = \mu_B B_0 l_i \left(\frac{\partial g_i}{\partial Q_\alpha}\right) \langle n_\alpha | \hat{Q}_\alpha | n'_\alpha \rangle \langle SM_S | \hat{S}_i | SM'_S \rangle$$

In practice, we define $g_i^\alpha$, $D^\alpha$, and $E^\alpha$ as following:

$$g_i^\alpha = \frac{\partial g_i}{\partial Q_\alpha} \langle 0_\alpha | \hat{Q}_\alpha | 1_\alpha \rangle$$

$$D^\alpha = \frac{\partial D}{\partial Q_\alpha} \langle 0_\alpha | \hat{Q}_\alpha | 1_\alpha \rangle$$

$$E^\alpha = \frac{\partial E}{\partial Q_\alpha} \langle 0_\alpha | \hat{Q}_\alpha | 1_\alpha \rangle$$

Under the harmonic oscillator approximation, the spin-phonon coupling matrix elements can be easily expressed with $g_i^\alpha$, $D^\alpha$, and $E^\alpha$.

We adopt this definition to avoid ambiguity associated with the definition of normal coordinates, which can be dimensionless or expressed in units of length. Consequently, the values of derivatives such as $\frac{\partial g_i}{\partial Q_\alpha}$, $\frac{\partial D}{\partial Q_\alpha}$, and $\frac{\partial E}{\partial Q_\alpha}$ vary depending on how the normal coordinates are defined. In contrast, the derived $g_i^\alpha$, $D^\alpha$, and $E^\alpha$ are independent of the specific definition of the normal coordinates. These parameters retain the same units as the conventional spin Hamiltonian parameters $g_i$, $D$, and $E$, making them a more intuitive and practical choice for experimental comparison.

## Explicit expression of the matrix elements of $\hat{H}_{tot}$ in $|\boldsymbol{v},SM_S\rangle$ basis

With the current extended spin Hamiltonian:

$$\hat{H}_{tot} = \hat{H}_S + \hat{H}_{Ph} + \hat{H}_{S-Ph}$$

$$\hat{H}_S = \sum_{i=x,y,z} \mu_B B_0 l_i g_i \hat{S}_i + D\left(\hat{S}_z^2 - \tfrac{1}{3}S(S+1)\right) + E\left(\hat{S}_x^2 - \hat{S}_y^2\right)$$

$$\hat{H}_{Ph} = \sum_\alpha^N \hbar\omega_\alpha\left(\hat{n}_\alpha + \tfrac{1}{2}\right)$$

$$\hat{H}_{S-Ph} = \sum_\alpha^N\left[\sum_{i=x,y,z} \mu_B B_0 l_i \left(\tfrac{\partial g_i}{\partial Q_\alpha}\right)\hat{S}_i + \left(\tfrac{\partial D}{\partial Q_\alpha}\right)\left(\hat{S}_z^2 - \tfrac{1}{3}S(S+1)\right) + \left(\tfrac{\partial E}{\partial Q_\alpha}\right)\left(\hat{S}_x^2 - \hat{S}_y^2\right)\right]\hat{Q}_\alpha$$

Assuming two phonons, the Hamiltonian matrix can be expressed as:

| | $\lvert\{0_\alpha,0_\beta\},SM_S\rangle$ | $\lvert\{1_\alpha,0_\beta\},SM_S\rangle$ | $\lvert\{0_\alpha,1_\beta\},SM_S\rangle$ | $\lvert\{1_\alpha,1_\beta\},SM_S\rangle$ |
|---|---|---|---|---|
| $\lvert\{0_\alpha,0_\beta\},SM_S\rangle$ | $\boldsymbol{H_S + H_{Ph,00}}$ | $\boldsymbol{H_{S-Ph,\alpha}}$ | $\boldsymbol{H_{S-Ph,\beta}}$ | $\boldsymbol{0}$ |
| $\lvert\{1_\alpha,0_\beta\},SM_S\rangle$ | $\boldsymbol{H_{S-Ph,\alpha}}$ | $\boldsymbol{H_S + H_{Ph,10}}$ | $\boldsymbol{0}$ | $\boldsymbol{H_{S-Ph,\beta}}$ |
| $\lvert\{0_\alpha,1_\beta\},SM_S\rangle$ | $\boldsymbol{H_{S-Ph,\beta}}$ | $\boldsymbol{0}$ | $\boldsymbol{H_S + H_{Ph,01}}$ | $\boldsymbol{H_{S-Ph,\alpha}}$ |
| $\lvert\{1_\alpha,1_\beta\},SM_S\rangle$ | $\boldsymbol{0}$ | $\boldsymbol{H_{S-Ph,\beta}}$ | $\boldsymbol{H_{S-Ph,\alpha}}$ | $\boldsymbol{H_S + H_{Ph,11}}$ |

In which

$$[\boldsymbol{H_S}]_{i,j} = \left\langle SM_{S,i}\,\middle|\,\hat{H}_S\,\middle|\,SM_{S,j}\right\rangle$$

$$\left[\boldsymbol{H_{Ph,n_\alpha n_\beta}}\right]_{i,j} = \left[\hbar\omega_\alpha\left(n_\alpha + \frac{1}{2}\right) + \hbar\omega_\beta\left(n_\beta + \frac{1}{2}\right)\right]\delta_{ij}$$

And the spin-phonon coupling matrix can be conveniently represented in terms of spin-phonon coupling parameters:

$$\left[\boldsymbol{H_{S-Ph,\alpha}}\right]_{i,j} = \left\langle SM_{S,i}\,\middle|\,\sum_{i=x,y,z}\mu_B B_0 l_i g_i^\alpha \hat{S}_i + D^\alpha\left(\hat{S}_z^2 - \frac{1}{3}S(S+1)\right) + E^\alpha\left(\hat{S}_x^2 - \hat{S}_y^2\right)\,\middle|\,SM_{S,j}\right\rangle$$

The spin Hamiltonian matrix and the phonon matrix form the block diagonal part in the vibrational quanta, while the spin-phonon coupling matrix acts as off-diagonal in the vibrational quanta and mixes different spin and vibrational levels.

In the current formalism, we consider only the linear (first-order) spin-phonon coupling term. While this operator directly couples states differing by a single vibrational quantum, it also indirectly couples states differing by multiple quanta. For instance, the vibrational ground state $|(0_\alpha 0_\beta),SM_S'\rangle$ directly couples to $|(0_\alpha 1_\beta),SM_S'\rangle$, which in turn couples to $|(1_\alpha 1_\beta),SM_S''\rangle$. Thus, to second order, even the first-order spin-phonon term enables mixing between states that differ by two vibrational quanta. This has parallels to the well-known Brillouin's theorem in quantum chemistry especially when used within a configuration interaction (CI) formalism that states that excited states with a configuration state

function (CSF) that differ by 1 electron from the ground state CSF do not directly couple to the ground-state CSF, but they do couple indirectly.

We can also explicitly write the general expression for the matrix elements of $\hat{H}_{tot}$ using spin ladder operators:

$$\left\langle \boldsymbol{v}', SM_S' \middle| \hat{H}_{tot} \middle| \boldsymbol{v}, SM_S \right\rangle =$$

$$\delta_{\boldsymbol{vv}'} \left[\frac{1}{2}\left(\delta_{M_S+1M_S'} + \delta_{M_SM_S'+1}\right)\mu_B B_0 l_x g_x \sqrt{S(S+1) - M_S M_S'} + \frac{1}{2}\left(\delta_{M_S+1M_S'} - \delta_{M_SM_S'+1}\right)\mu_B B_0 l_y g_y \sqrt{S(S+1) - M_S M_S'} + \delta\right.$$

$$+ \delta_{\boldsymbol{vv}'} \delta_{M_SM_S'} \sum_\alpha^N \hbar\omega_\alpha \left(n_\alpha + \frac{1}{2}\right)$$

$$+ \sum_\alpha^N \left(\delta_{\ldots v_\alpha \ldots, \ldots v'_\alpha \pm 1 \ldots}\right)\left[\frac{1}{2}\left(\delta_{M_S+1M_S'} + \delta_{M_SM_S'+1}\right)\mu_B B_0 l_x g_x^\alpha \sqrt{S(S+1) - M_S M_S'} + \frac{1}{2}\left(\delta_{M_S+1M_S'} - \delta_{M_SM_S'+1}\right)\mu_B B_0 l_y g_y^\alpha \sqrt{S(\right.$$

## Unit conversion of spin-phonon coupling parameters

In ab initio calculations, the parameters are typically given in atomic units. They need to be converted to the conventional units of spin Hamiltonian parameters. Here, we provide one example to show how to conduct such unit conversion.

In our ab initio calculations using ORCA, we use normal coordinates with the unit of length. Assume the $\frac{\partial D}{\partial Q_\alpha}$ has the unit of (Hartree/Bohr), and the vibrational frequency has the unit of ($cm^{-1}$), and the reduced mass (since we didn't use the dimensionless definition) has the unit of (amu):

$$\frac{\partial D}{\partial Q_\alpha} = a\left(\frac{Hartree}{Bohr}\right)$$

Vibrational frequency: $\omega_\alpha = 2\pi c \cdot \tilde{v}_\alpha$, and $\tilde{v}_\alpha = b\ (cm^{-1})$, where $c$ is the velocity of light.

Reduced mass: $m = m_\alpha\ (amu)$

Since $\langle 0_\alpha | \hat{Q}_\alpha | 1_\alpha \rangle = \sqrt{\frac{\hbar}{2m\omega_\alpha}}$ and we need $\frac{\partial D}{\partial Q_\alpha}\langle 0_\alpha | \hat{Q}_\alpha | 1_\alpha \rangle$, we have:

$$D^\alpha = \frac{\partial D}{\partial Q_\alpha}\langle 0_\alpha | \hat{Q}_\alpha | 1_\alpha \rangle = a\left(\frac{Hartree}{Bohr}\right) \cdot \sqrt{\frac{\hbar}{2m\omega_\alpha}}$$

$$= a\left(\frac{Hartree}{5.29\times10^{-11}\ (m)}\right) \cdot \sqrt{\frac{1.05\times10^{-34}\ (J\cdot s)}{2\cdot m_\alpha \cdot 1.66\times10^{-27}\ (kg)\cdot 2\times3.14\times3\times10^{8}\ (m\cdot s^{-1})\cdot b\ (cm^{-1})}}$$

$$= 7.74 \times a\sqrt{\frac{1}{m_\alpha b}}\ (Hartree)$$

$$= a\sqrt{\frac{1}{m_\alpha b}} \cdot (1.7 \times 10^{6})\ (cm^{-1})$$

If the unit of $\frac{\partial D}{\partial Q_\alpha}$ is $cm^{-1}$/Å, then the expression becomes:

$$D^\alpha = 4.095 \times a\sqrt{\frac{1}{m_\alpha b}}\ (cm^{-1})$$

# Raw Experimental Data

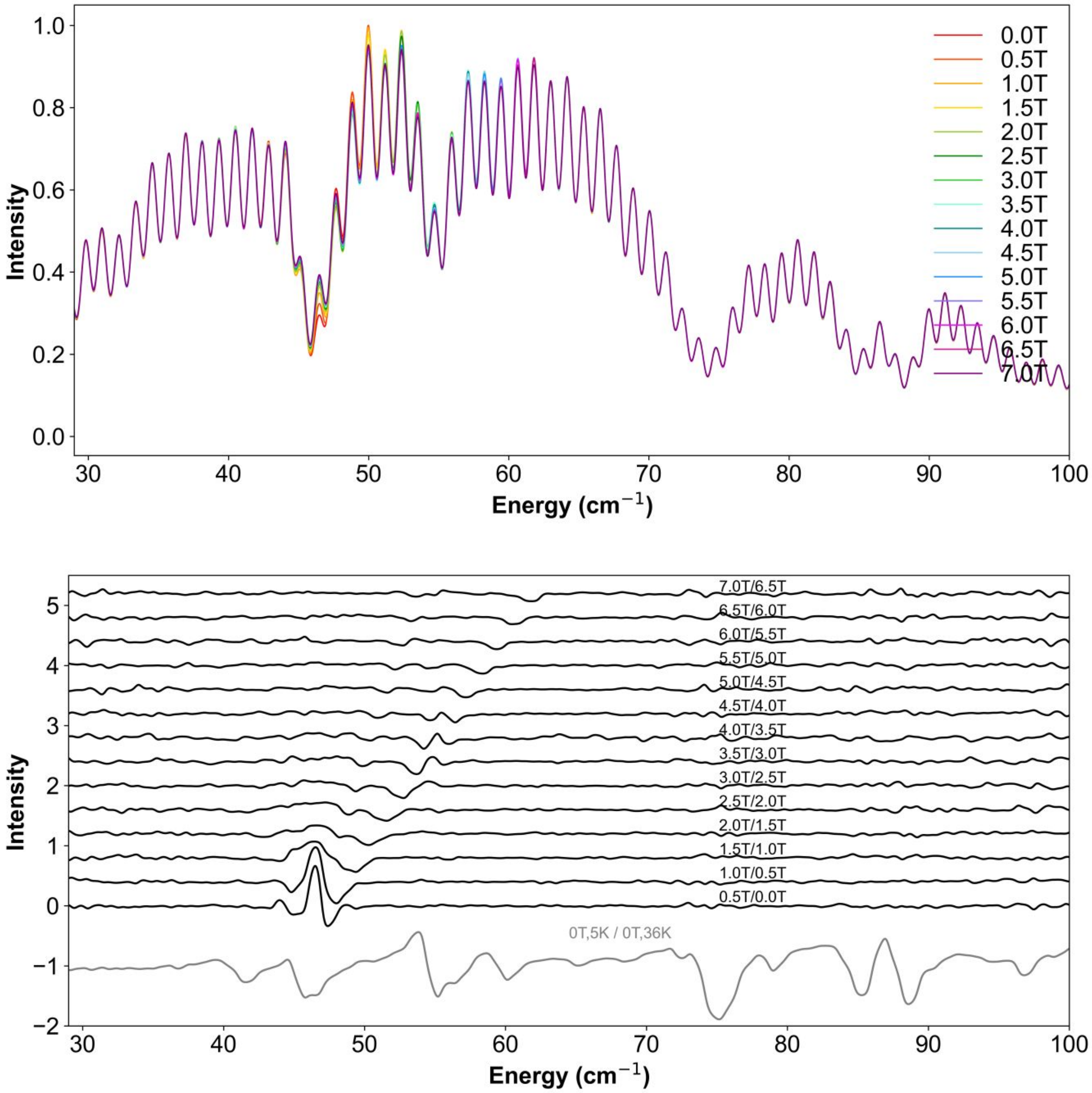


**Figure S1.** (top) The raw full transmission spectra. (bottom) The full magnetic field division spectra (black curves) and the division spectrum of transmittance at 5 K by that at 36 K under 0 T (grey).

# High-frequency filtration of raw transmission data

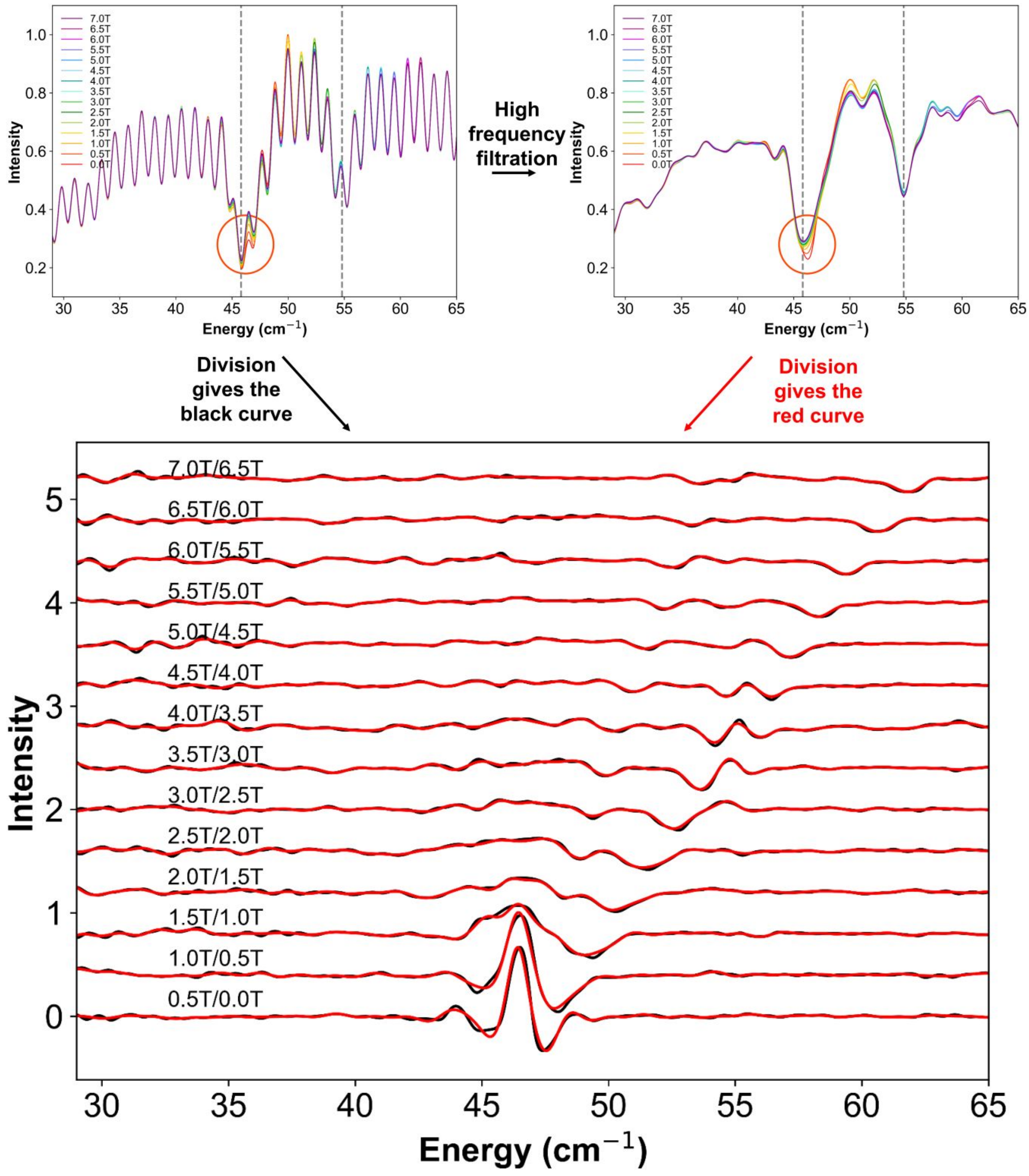


**Figure S2.** Overlay of field-division spectra of raw transmission spectra (black) and high-frequency filtered spectra (red).

The main spectral features are preserved, which further lends credence to this high-frequency filtration process of removing the oscillation background. Even so, the field-division spectra used in the main text are derived from the unfiltered transmission data.

The cutoff of the frequency filtration is 0.7 “Hz”. The unit here is not real Hz but the reverse of the actual unit (cm$^{-1}$) of the energy axis used here, which has the formal unit of:

$$\frac{1}{hc\tilde{\nu}} = \frac{1}{6.626 \times 10^{23} \times J \cdot s \times \ 3 \times 10^{8} \times m \cdot s^{-1} \times 1 \times cm^{-1}} \approx 5 \times 10^{-35} \, J^{-1}$$

For practical completeness, the following python script is used to process this signal filtration:

```
import numpy as np
import matplotlib.pyplot as plt
import matplotlib.patches as patches
data = np.loadtxt("experimental.txt")
energy = data[:,0]
spc = data[:,1:]
size = energy.size

fig = plt.figure(figsize = (12,6))
ax1 = fig.add_subplot(1,2,1)
ax2 = fig.add_subplot(1,2,2)

filter_spc = np.zeros((size,15))
cutoff = 0.7
for i in range(15):
    fft_data = np.fft.fft(spc[:,i])
    freq = np.fft.fftfreq(len(spc[:,i]), d=(energy[1] - energy[0]))  # Frequency axis
    # Filter: keep only high frequencies (e.g., abs(freq) > cutoff)
    fft_filtered = fft_data.copy()
    fft_filtered[np.abs(freq) > cutoff] = 0
    # Inverse FFT
    filter_spc[:,i] = np.fft.ifft(fft_filtered).real
    graph1 = ax1.plot(energy,spc[:,i],label=str(i/2)+'T',color=color_bar[i],linewidth = 1.0)
    graph1 = ax2.plot(energy,filter_spc[:,i],label=str(i/2)+'T',color=color_bar[i],linewidth = 1.0)

ax1.set_xlabel('Energy (cm$^{-1}$)',fontsize=18, fontweight='bold', fontfamily='Arial')
ax1.set_ylabel('Intensity',fontsize=18, fontweight='bold', fontfamily='Arial')
ax1.set_xlim(29,65)
# ax1.set_ylim(0.1,0.6)
handles, labels = ax1.get_legend_handles_labels()
# ax1.legend(handles[::-1], labels[::-1], loc='lower right')
# ax1.legend(handles[::-1], labels[::-1], loc='lower right',fontsize=18, labelspacing=0.9, frameon=False)
ax2.set_xlabel('Energy (cm$^{-1}$)',fontsize=18, fontweight='bold', fontfamily='Arial')
ax2.set_ylabel('Intensity',fontsize=18, fontweight='bold', fontfamily='Arial')
ax2.set_xlim(29,65)
# ax2.set_ylim(0.1,0.6)
handles, labels = ax2.get_legend_handles_labels()
# ax2.legend(handles[::-1], labels[::-1], loc='lower right')
# ax2.legend(handles[::-1], labels[::-1], loc='lower right',fontsize=18, labelspacing=0.9, frameon=False)

plt.tight_layout()
# plt.savefig('field_dependence2_ 20241015.png', format='png',dpi = 600)
plt.show()
```

# Additional simulations

## Varying spin-phonon coupling strength and phonon transition intensity

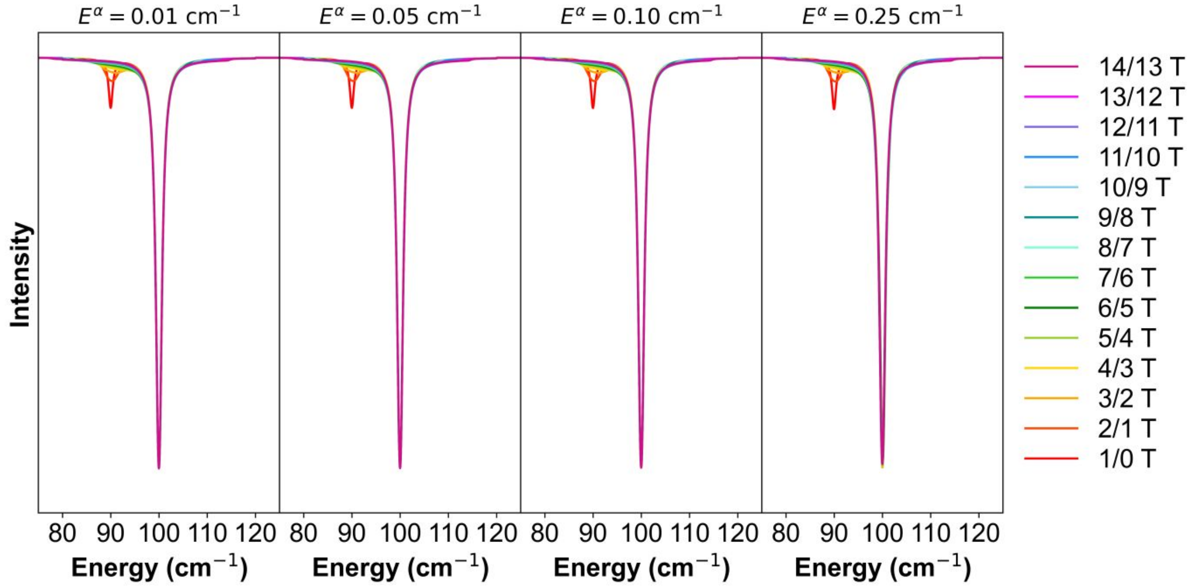


**Figure S3.** Transmission spectra with systematically varied spin-phonon coupling parameter $E^{\alpha}$ from 0.01 to 0.25 cm$^{-1}$.

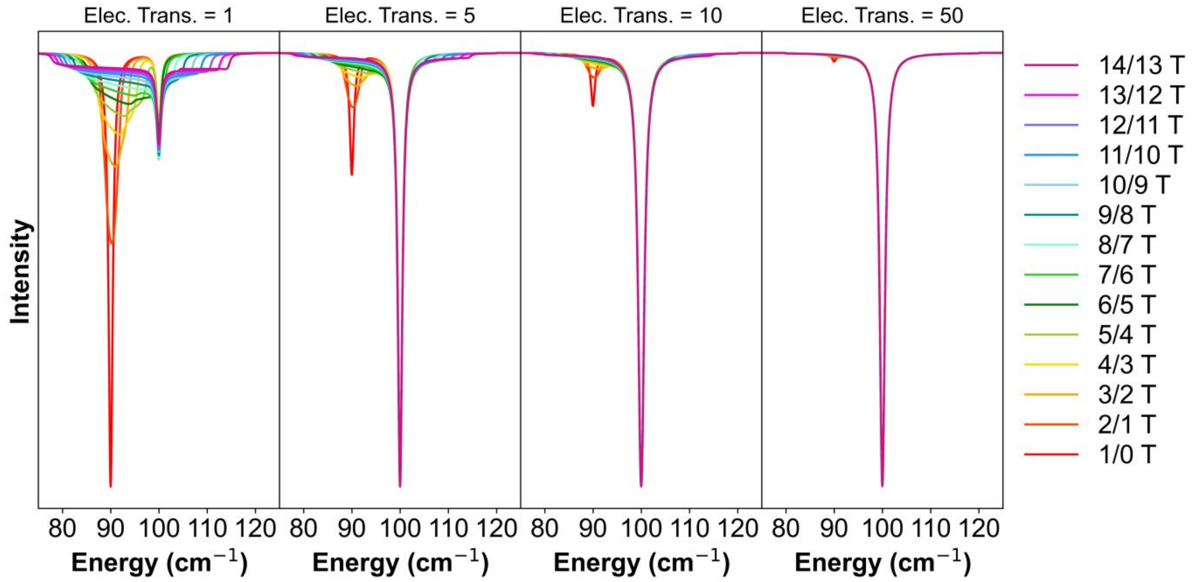


**Figure S4.** Transmission spectra with systematically varied phonon intensity with the $E^{\alpha}$ fixed at 0.1 cm$^{-1}$.

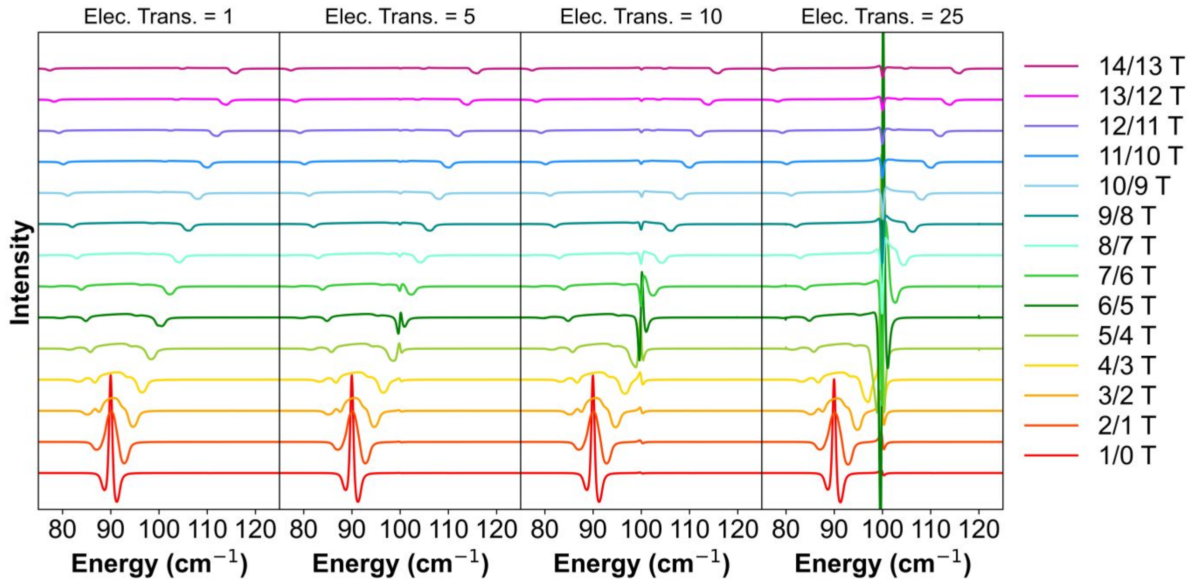


**Figure S5.** Field-division spectra with systematically varied phonon intensity with the $E^{\alpha}$ fixed at 0.1 cm$^{-1}$.

## Orientation of the electric transition dipole moment

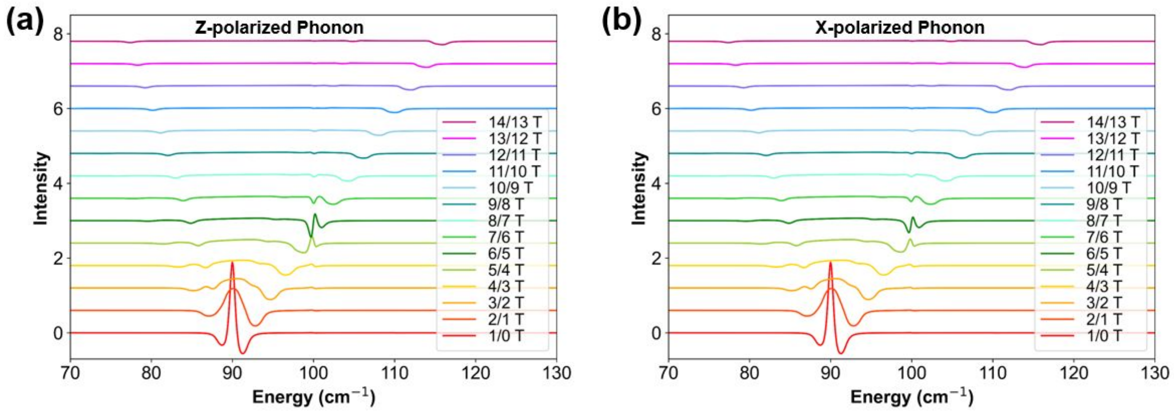


**Figure S6.** Influence of the phonon polarization direction to the powder transmission and division spectra. (a) The field-division of z-polarized phonon absorption. (b) The field-division of x-polarized phonon absorption. $S$ = 3/2, $D$ = 45 cm$^{-1}$, $E/D$ = 0, ($g_x$, $g_y$, $g_z$) = (2, 2, 2), phonon absorption at 100 cm$^{-1}$, $E^{\alpha}$= 0.05 cm$^{-1}$, FWHM = 1 cm$^{-1}$. Voigt mode.

As is seen, Voigt mode (with powder average) is not very sensitive to the polarization direction of the phonon.

## The contribution of $g^{\alpha}_{x/y}$

$g^{\alpha}_{x}$ and $g^{\alpha}_{y}$ serve as off-diagonal elements in the spin-phonon coupling matrix and connect levels with $\Delta M_S = \pm1$. In an $S = 3/2$ system, when an EPR spectral branch crosses the phonon, this coupling could redistribute intensity between the EPR transition and the phonon, generating a second-derivative-like structure in the division spectra, similar to that induced by $E^{\alpha}$. Under Voigt mode, $g^{\alpha}_{x}$ and $g^{\alpha}_{y}$ contribute equally in an axially symmetric system. Unlike $E^{\alpha}$, the magnitude of the off-diagonal elements is field-dependent and grows with increasing field strength. Consequently, the $g^{\alpha}_{x/y}$-induced spin-phonon structures do not diminish rapidly at higher fields. In fact, at sufficiently large fields it evolves from a second-derivative-type to a first-derivative-type shape. As illustrated in Figure S7, with $g^{\alpha}_{x} = 0.1$, a clear second-derivative structure appears at 5T/4T; by 16 T this fully transforms into a first-derivative-like feature, whose amplitude continues to increase at higher fields.

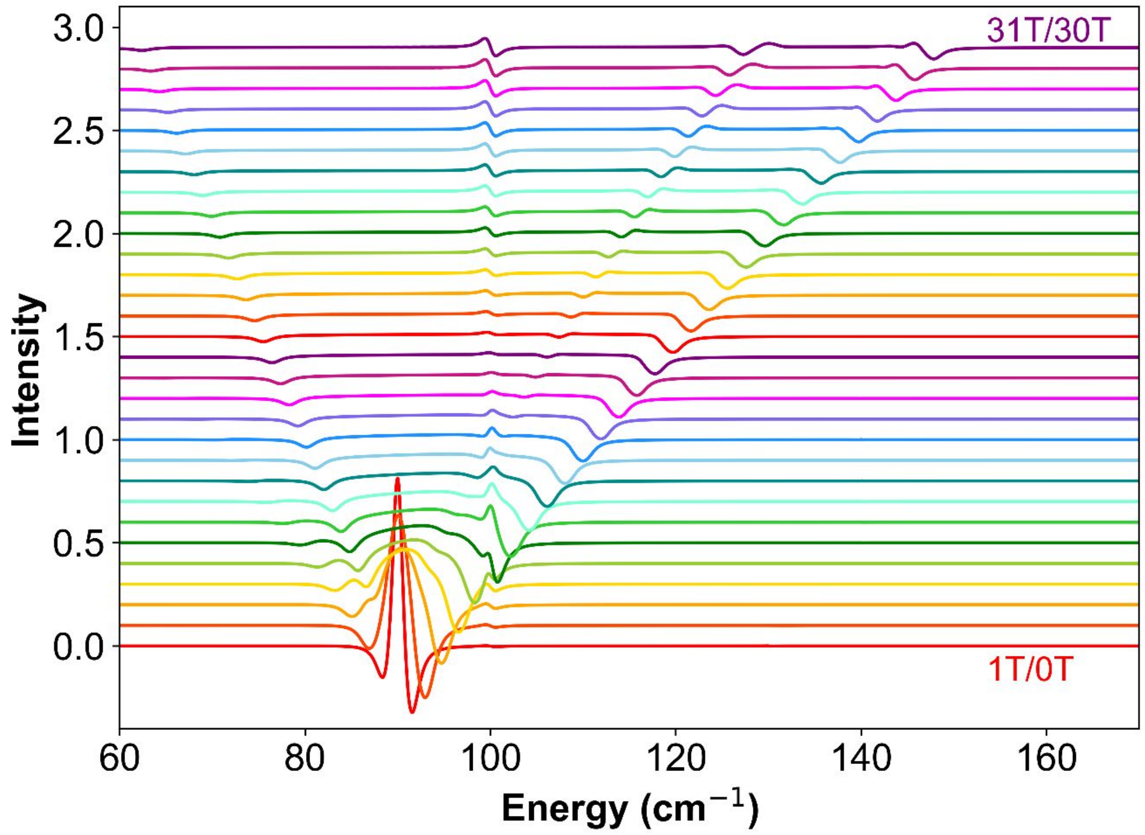


**Figure S7.** Field-division spectra of the one-phonon model. $g^{\alpha}_{x}$ = 0.1, FWHM = 2 cm$^{-1}$. The field varies from 0 to 30 T with a 1 T interval.

## The contribution of $D^{\alpha}$ and $g^{\alpha}_{z}$

Since $D^{\alpha}$ and $g^{\alpha}_{z}$ represent diagonal elements in the spin-phonon coupling matrix, they are unable to induce direct mixing between different Ms levels. Instead, they contribute via indirect mechanisms in the orientation average. Consequently, to produce an observable spin-phonon coupling signature in

the field-division spectra, their magnitudes must be comparable to or greater than the spectral linewidth. As illustrated in Figure S8(a), a value of $D^{\alpha}$ = 2.0 cm$^{-1}$ can induce a second-derivative-like spectral feature similar to that produced by $E^{\alpha}$, despite the latter spin-phonon coupling parameter being one to two orders of magnitude smaller. This second-derivative feature gradually evolves into a first-derivative-like structure, and as opposed to $g_{x/y}^{\alpha}$, its intensity is relatively field-independent.

A comparable scenario applies to $g_z^{\alpha}$. It requires a substantially larger magnitude than $g_{x/y}^{\alpha}$ to generate discernible features in the field-division spectra. As shown in Figure S8(b), even when $g_z^{\alpha}$ = 0.3, the spin-phonon coupling features are barely detectable at lower fields. Upon increasing the field to 10 T—corresponding to a Zeeman energy ($\mu_B g_z^{\alpha} B$) of approximately 1.4 cm$^{-1}$, comparable to the spectral linewidth—a weak first-derivative feature emerges, and its intensity increases upon elevating to higher field.

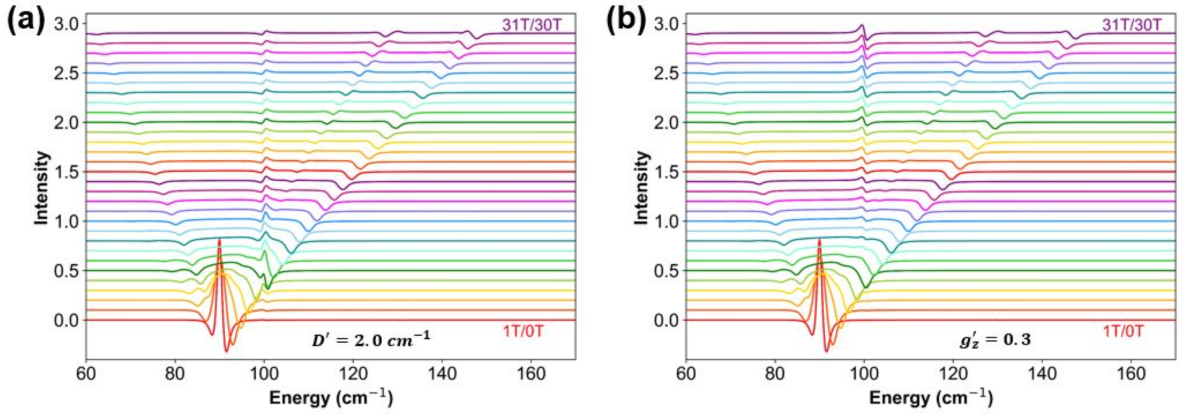


**Figure S8.** Field-division spectra of the one-phonon model with the field varies from 0 to 30 T with 1 T interval. (a) $D^{\alpha}$ = 2 cm$^{-1}$, FWHM = 2 cm$^{-1}$. (b) $g_Z^{\alpha}$ = 0.3, FWHM = 2 cm$^{-1}$.

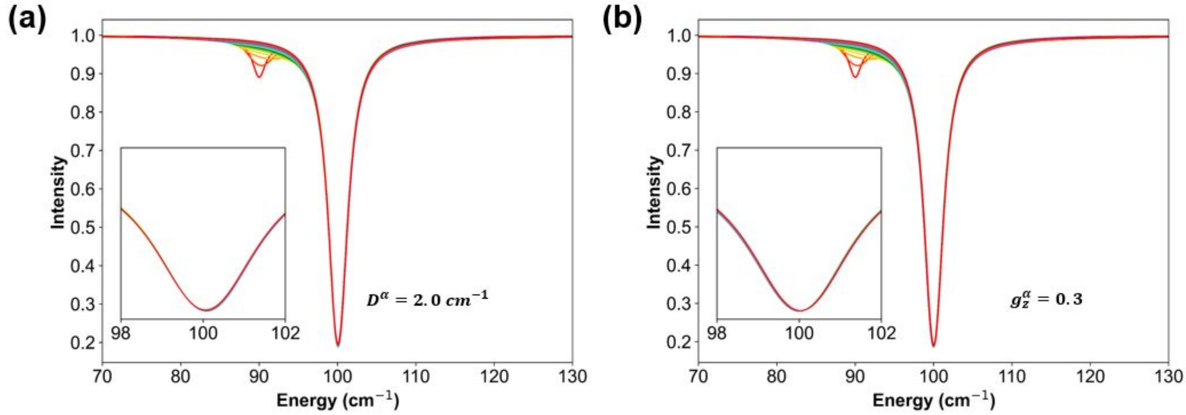


**Figure S9.** Transmission spectra of the one-phonon model with the magnetic field varying from 0 to 30 T with 1 T interval. (a) $D^{\alpha}$ = 2 cm$^{-1}$, FWHM = 2 cm$^{-1}$. (b) $g_z^{\alpha}$ = 0.3, FWHM = 2 cm$^{-1}$. No discernible splitting can be observed in the phonon absorption at 100 cm$^{-1}$.

## Sign of zero-field splitting

In principle, THz EPR can be used to determine the sign of the zero-field splitting parameter *D*, because reversing the sign of *D* effectively inverts the order of the two Kramers doublets. While the transition probability for a given orientation and transition remains unchanged, the corresponding energy splittings vary at a fixed temperature and magnetic field. Consequently, the evolution of the spectral branches differs depending on the sign of *D*. As illustrated below, by reversing the *D*-sign of the one-phonon model, the spectral branch evolves differently.

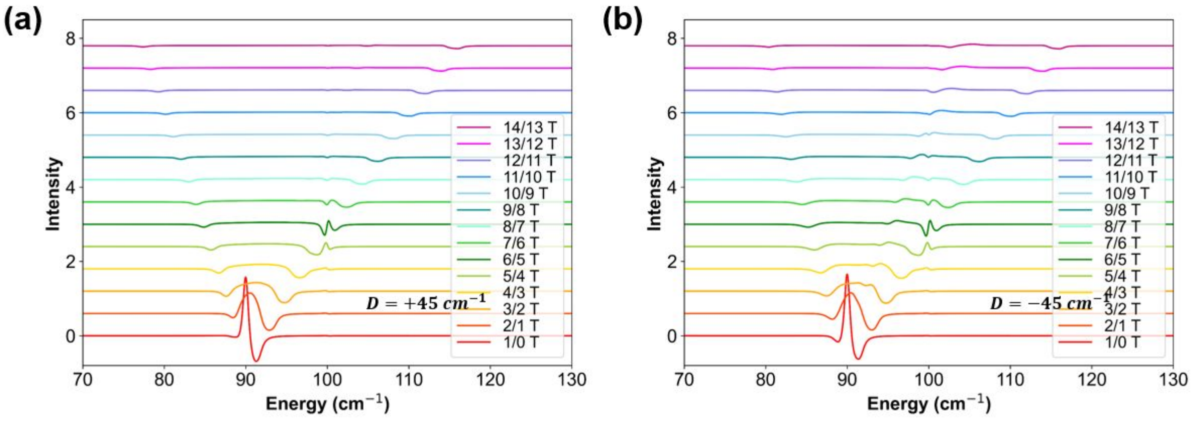


**Figure S10.** Field division spectra of the one-phonon model varies from 0 to 14 T with 1 T interval, with (a) a positive *D* of +45 cm$^{-1}$, and (b) a negative *D* of −45 cm$^{-1}$. Temperature fixed at 1 K.

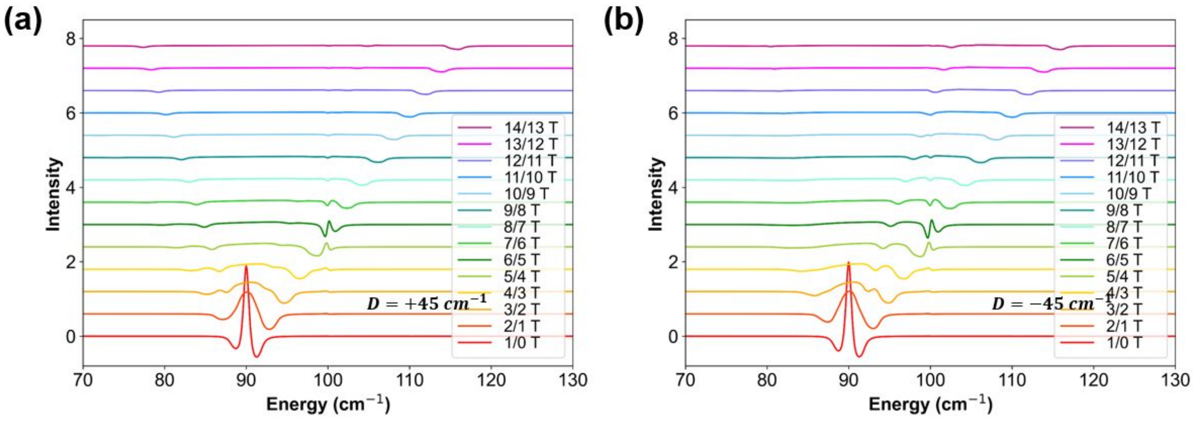


**Figure S11.** Field division spectra of the one-phonon model varies from 0 to 14 T with 1 T interval, with (a) a positive *D* of +45 cm$^{-1}$, and (b) a negative *D* of -45 cm$^{-1}$. Temperature fixed at 5 K.

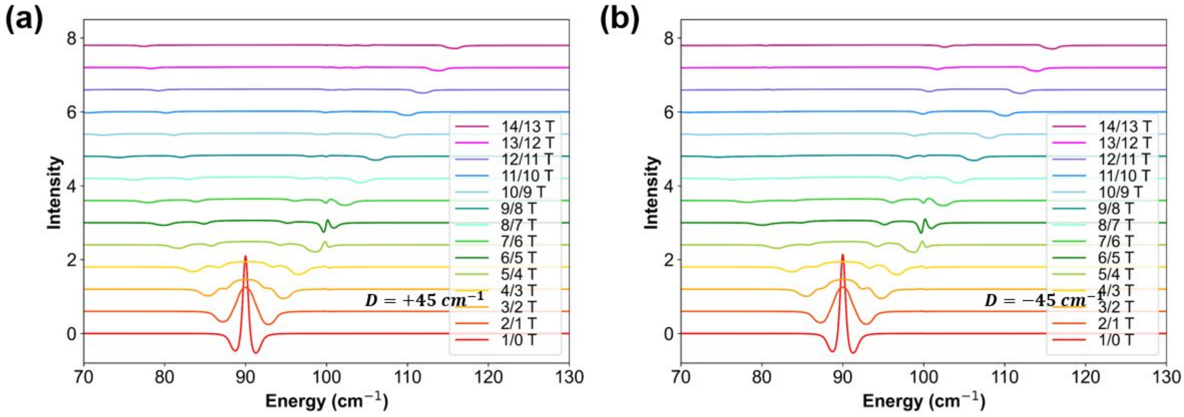


**Figure S12.** Field division spectra of the one-phonon model varies from 0 to 14 T with 1 T interval, with (a) a positive *D* of +45 cm$^{-1}$, and (b) a negative *D* of -45 cm$^{-1}$. Temperature fixed at 20 K.

An independent approach to determine the sign of *D* is to simulate the field-division spectra of the complex **1** using both positive and negative *D* values. As shown below, simulations based on a positive *D* value exhibit greater deviation from the experimental spectra compared to those using a negative *D*. Furthermore, a positive *D* value is inconsistent with the expected electronic structure of an elongation-distorted tetrahedral Co(II) complex, further supporting the assignment of a negative *D* in this system.

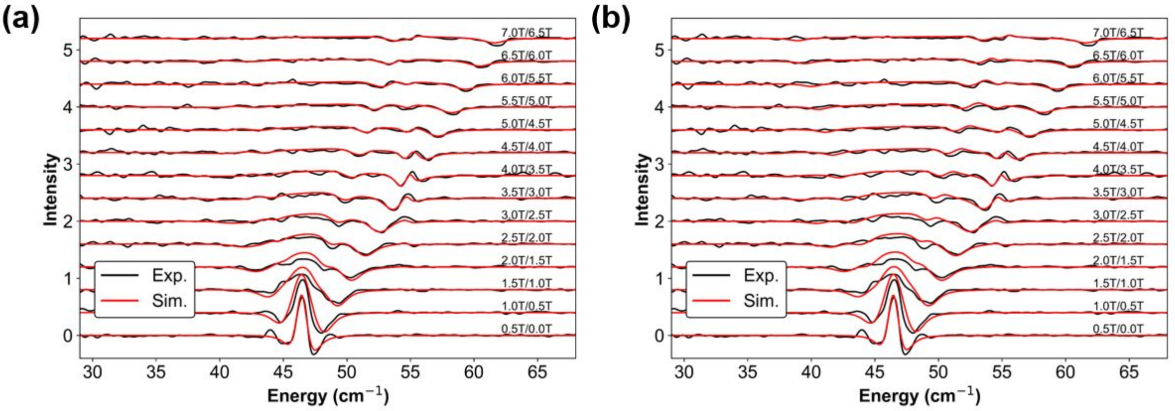


**Figure S13.** Simulation of field division spectra of the complex **1** with (left) the negative *D* of -23 cm$^{-1}$, and (right) the positive *D* of +23 cm$^{-1}$.

Another potential concern arises from the presence of multiple phonon or vibrational features in the transmission data, specifically, the minor "interference-like" structures around 44 and 46 cm$^{-1}$. To be honest, whether these correspond to genuine spin-phonon coupling features remains uncertain; therefore, only the most confident assignments are discussed in the main text.

The signal-to-noise ratio (S/N) of field-division spectra is primarily determined by the overall transmittance. When intense phonons are present, the detector can approach saturations, significantly reducing the S/N in those regions of the division spectra. In practice, this requires

balancing sample amount, sufficiently enough to resolve field-dependent spin-flip transition, yet not overload to avoid phonon saturation.

In our measurements, the transmittance was carefully maintained above 0.2 (relative to the highest transmitted points in the normalized spectrum). Features exceeding three times the baseline noise (e.g. near 35 $cm^{-1}$) were considered as faithful signals. The small feature near 46 $cm^{-1}$, particularly visible in the 2.0 T / 1.5 T and 1.5 T / 1.0 T division spectra, lies close to this threshold and is therefore difficult to unambiguously classify as signal or noise.

Typically, the lowest-field THz EPR division spectra exhibit a negative-positive-negative, second-derivative-like pattern. However, in our case, the observed structure first increases in clearly exceeds the noise level. This feature cannot be adequately simulated using the 45.8 $cm^{-1}$ phonon and appears to require an additional, weaker mode around 44 $cm^{-1}$. Although a subtle bulge at this frequency is present in the filtered transmission spectrum, its intensity is marginal, and we cannot confidently conclude that we must introduce this weak phonon in the simulation.

## Theoretical Model of $[CoCl_4]^{2-}$

We use this high-spin $d^7$ $[CoCl_4]^{2-}$ complex of idealized symmetry to rationalize the spin-phonon coupling pattern in complex **1**. Unlike other octahedral complexes, tetrahedral high spin $d^7$ configuration has no Jahn-Teller distortion and therefore, the optimization at DFT level, with the single reference framework, can effectively describe their geometric structures and distortions induced by the ligand scaffold.

$[CoCl_4]^{2-}$ complex is highly symmetric and exhibits nine vibrational modes: symmetric and asymmetric bending and stretching (Figure 11). The symmetric bending modes are doubly degenerate; the scissoring mode lowers the symmetry to $S_4$, while the twisting mode lowers it to $D_2$, as shown in Figure S14. In both cases, the irreducible representation degrades from E in $T_d$ point group to A in the reduced symmetry groups. The symmetric stretching mode is the simplest, involving in-phase radial displacement of all ligands, which preserves the overall symmetry. By contrast, the asymmetric bending and stretching modes are triply degenerate, decomposable into three motions where the metal center is displaced along x, y, or z axis. These distortions reduce the symmetry from $T_d$ to $C_{2v}$, with the irreducible representation correspondingly degrading to A. Among these modes, the symmetric bending has the lowest frequency, while the asymmetric stretching has the highest.

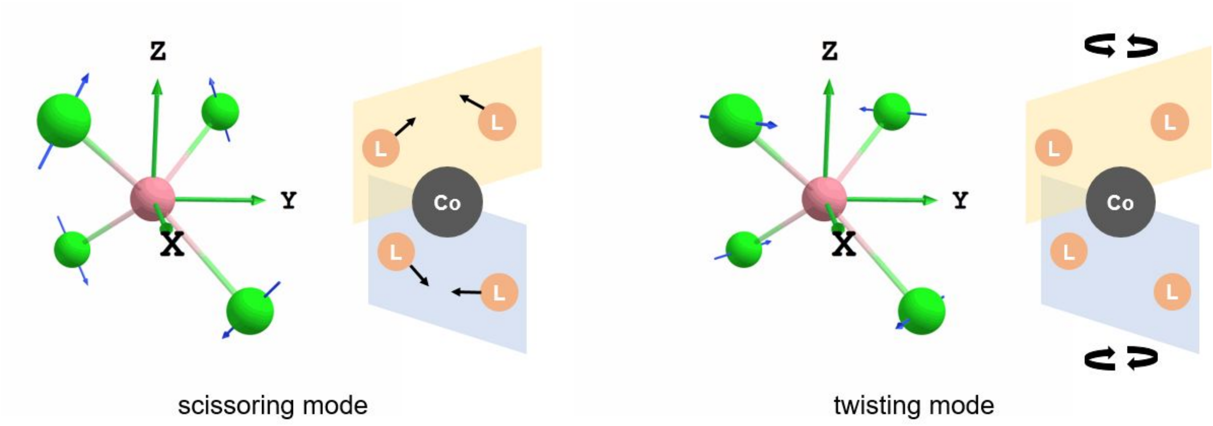


**Figure S14.** The scissoring and twisting modes.

Among these vibrational modes, the two symmetric bending modes are particularly important for rationalizing the spin-phonon coupling pattern in complex **1**, in which their AILFT results and D-tensor along the normal coordinates are shown in Figure S15. We analyze the magnetic properties as functions of vibrational motion, where the linear spin-phonon coupling terms correspond to the first derivatives of these normal modes. In Werner-type 3d transition-metal systems, such coupling mainly arise from vibrational modulation of the five 3d orbital energy levels, which can be intuitively understood using ligand field theory.

The scissoring mode primarily alters the angular overlap between the ligands of $d_{xz}/d_{yz}$ orbitals and maintains the degeneracy of $d_{xz}/d_{yz}$. AILFT provides a quantitative way to visualize this modulation. During vibration, elongation leads to the easy-plane magnetization, while compression causes easy-axis magnetization, resulting in a reversal of the sign of $D$ value at equilibrium. Because the axial symmetry is preserved, this mode yields a large $D^{\alpha}$ parameter and rigorously zero $E^{\alpha}$.

The twisting mode, by contrast, lowers the axial symmetry and introduces rhombicity. It also modulates the overlap between ligands and the $d_{xz}/d_{yz}$ orbitals but induces a large energy splitting

between them. While the spin Hamiltonian formalism remains valid, this mode produces an apparent "discontinuity" in the *D* value at equilibrium due to a switch in the principal axes of the D-tensor when following the conventional order of $|D_z|>|D_y|>|D_x|$. In such cases, the overall *D*-tensor remains continuous, its derivative is diagonal, and spin-phonon coupling parameters can still be consistently defined. This vibrational mode gives rise to non-zero $D^{\alpha}$ and $E^{\alpha}$ parameters.

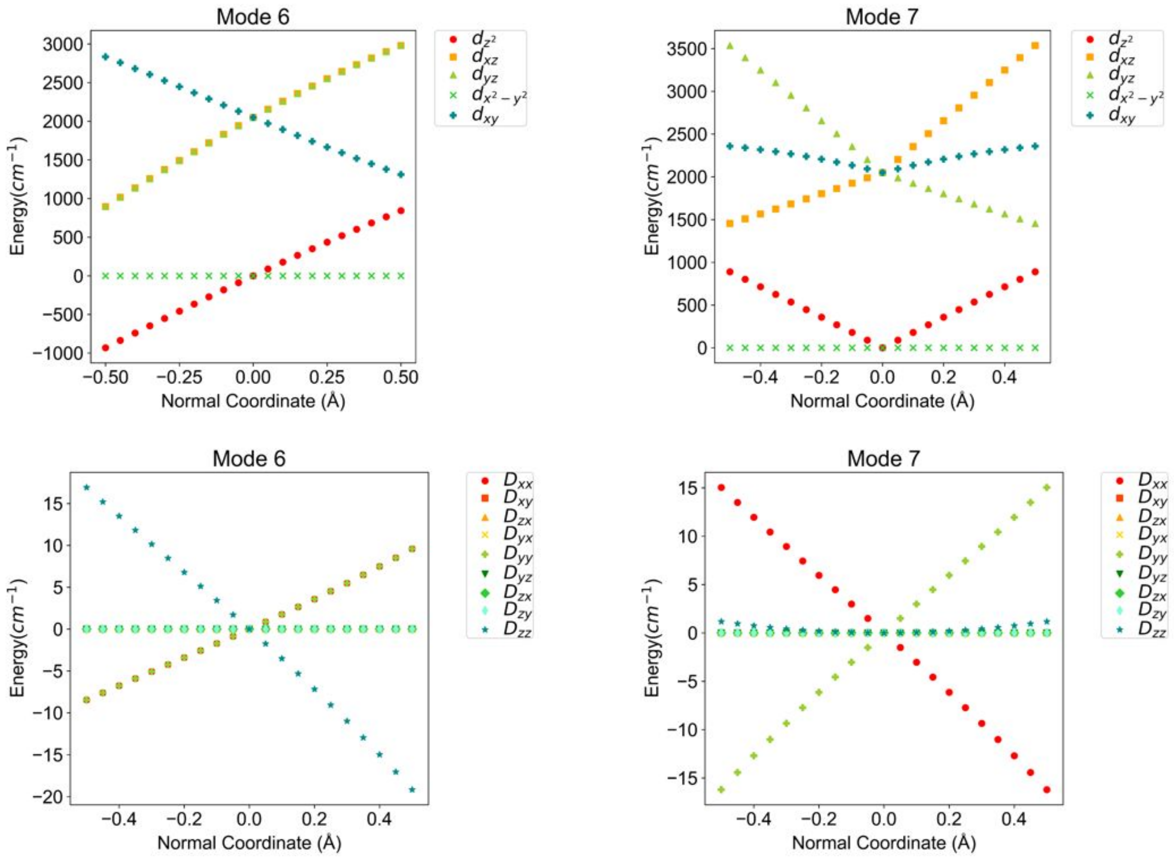


**Figure S15.** AILFT (top) and *D*-tensor (bottom) of the scissoring mode (left) and twisting mode (right).

The effect of the symmetric stretching mode requires clarification: in this complex it does not contribute to the ZFS. In other systems with non-zero ZFS, however, this mode would scale the ligand field strength and modulate the ZFS parameters, yielding finite spin-phonon coupling parameters. Though this coupling is expected to be much smaller than couplings induced by other vibrational modes.

The asymmetric bending and stretching modes give rise to a slightly different *D*-tensor orientation, with three principal axes rotated by ~45° relative to those of the symmetric bending. This arises because these modes effectively align the local x and y axes within the Cl-Co-Cl planes. Aside from this reorientation, the principal axes remain unchanged. If, however, the equilibrium structure already exhibits non-zero ZFS and the principal axes of the D-tensor are not aligned with the Cl-Co-Cl planes, these asymmetric modes may additional induce a rotation of the principal axes.

The frequencies of these modes and corresponding calculated spin-phonon coupling parameters are summarized in Table S1.

**Table S1.** Vibrational frequencies and calculated spin-phonon coupling parameters of normal modes in $[CoCl_4]^{2-}$ complex.

| Mode | | Vibrational frequency $(cm^{-1})$ | $\|D^{\alpha}\|$ $(cm^{-1})$ | $\|E^{\alpha}\|$ $(cm^{-1})$ |
|---|---|---|---|---|
| Symmetric bending | Scissoring mode | 73 | 4.20 | 0.00 |
| | Twisting mode | 73 | 3.68 | 1.17 |
| Asymmetric bending | | 121 | 0.89 | 0.19 |
| Symmetric stretching | | 244 | 0.00 | 0.00 |
| Asymmetric stretching | | 280 | 4.47 | 1.11 |

# Theoretical Model of $[Co(ndh)_2]^{2-}$

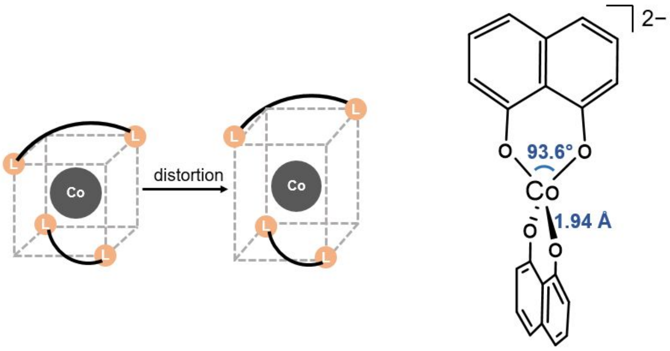


**Figure S16.** Schematic diagram of the distortion and structure of $[Co(ndh)_2]^{2-}$.

To incorporate the linkage, we employed a relatively symmetric ligand, naphthalene-1,8-diol (ndh), which induces an elongated distortion relative to the ideal $T_d$ structure (Figure S16). This distortion destabilizes the $d_{xz}/d_{yz}$ orbitals while stabilizing the $d_{xy}$ orbital. For the quartet multiplicity, there are in total ten roots within the 3d manifold. The tetrahedral coordination results in a ground state configuration of $(d_{z2})^2(d_{x2-y2})^2(d_{xy})^1(d_{xz})^1(d_{yz})^1$. The subsequent roots are summarized in Table S2.

Due to this distortion, the complex exhibits finite zero-field splitting (ZFS). Among the excited states, three low-lying ones contribute most significantly to ZFS (Figure S17). The lowest excited state is dominated by the configuration $(d_{z2})^2(d_{x2-y2})^1(d_{xy})^2(d_{xz})^1(d_{yz})^1$, which can be described as a single-electron excitation from $d_{x2-y2}$ to $d_{xy}$. Out-of-state spin-orbit coupling (SOC) between this state and the ground state generates unquenched orbital angular momentum along the molecular z axis, leading to a positive *g*-shift in this direction and a dominant negative contribution to ZFS. The next two excited states correspond to excitations from $d_{z2}$ to $d_{xz/yz}$, SOC with the ground state introduces unquenched orbital angular momentum along the molecular x and y axes. Because the second order mixing induced by SOC roughly scales inversely with the excitation energy, these states yield smaller *g*-shifts in the x/y directions and account for the rhombicity parameter.

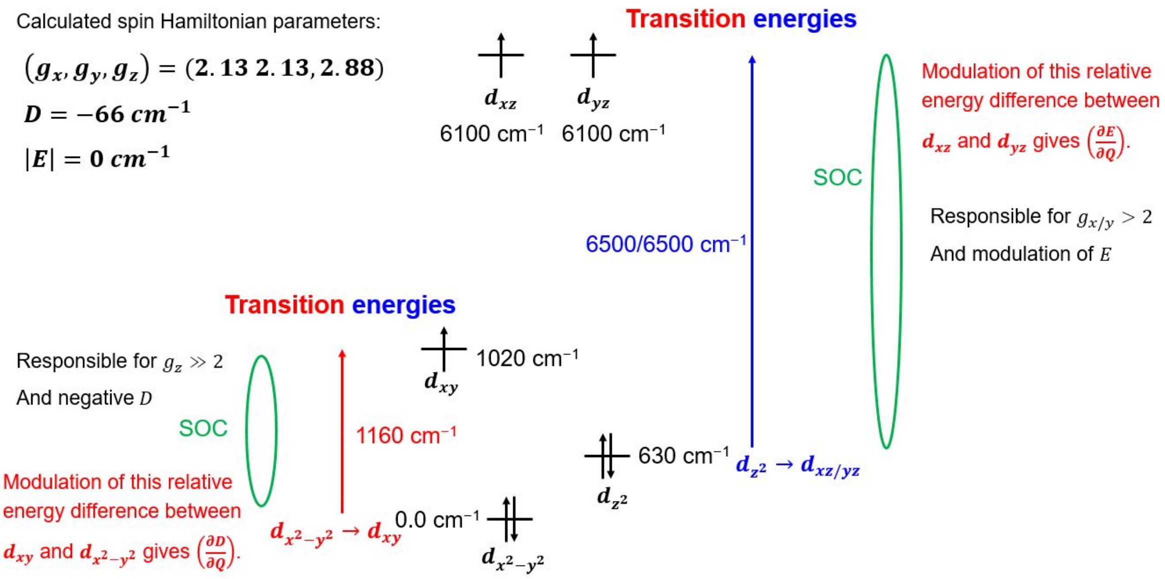

**Figure S17.** AILFT orbital energies (black) and key excited states with transition energies (red and blue) of $[Co(ndh)_2]^{2-}$.

**Table S2.** Calculated energies and dominant configuration state function (CSF) weights (larger than 0.2) of each root. The orbital sequence is displayed as $d_{z2}$, $d_{xz}$, $d_{yz}$, $d_{x2-y2}$, $d_{xy}$, 21121 denotes the occupation number of that CSF is $(d_{z2})^2(d_{xz})^1(d_{yz})^1$ $(d_{x2-y2})^2(d_{xy})^1$.

| Root | Energy (cm$^{-1}$) | | weight | CSF | Translation |
|---|---|---|---|---|---|
| | CASSCF | NEVPT2 | | | |
| 0 | 0 | 0 | 1.0000 | 21121 | Ground state |
| 1 | 1160 | 1160 | 1.0000 | 21112 | Single excitation:<br>$d_{x2-y2} \rightarrow d_{xy}$ |
| 2 | 5230 | 6500 | 0.59989<br>0.21716 | 12121<br>11221 | Single excitation:<br>$d_{z2} \rightarrow d_{xz/yz}$ |
| 3 | 5230 | 6500 | 0.59989<br>0.21716 | 11221<br>12121 | Single excitation:<br>$d_{z2} \rightarrow d_{xz/yz}$ |
| 4 | 6170 | 7350 | 0.43701<br>0.28593 | 12112<br>11212 | Double excitation:<br>$d_{x2-y2} \rightarrow d_{xy}$<br>$d_{z2} \rightarrow d_{xz/yz}$ |
| 5 | 6170 | 7350 | 0.43701<br>0.28593 | 11212<br>12112 | Double excitation:<br>$d_{x2-y2} \rightarrow d_{xy}$<br>$d_{z2} \rightarrow d_{xz/yz}$ |
| 6 | 7350 | 8170 | 0.53386<br>0.46614 | 11122<br>12211 | Single excitation:<br>$d_{z2} \rightarrow d_{xy}$<br>and<br>Double exciation:<br>$d_{x2-y2} \rightarrow d_{xy}$<br>$d_{z2} \rightarrow d_{xz/yz}$ |
| 7 | 21760 | 20240 | 0.53386<br>0.46614 | 12211<br>11122 | Single excitation:<br>$d_{z2} \rightarrow d_{xy}$<br>and<br>Double exciation:<br>$d_{x2-y2} \rightarrow d_{xy}$<br>$d_{z2} \rightarrow d_{xz/yz}$ |
| 8 | 24120 | 22170 | 0.41470<br>0.21068 | 22111<br>21211 | Single excitation:<br>$d_{x2-y2} \rightarrow d_{xz/yz}$ |
| 9 | 24120 | 22170 | 0.41470<br>0.21068 | 21211<br>22111 | Single excitation:<br>$d_{x2-y2} \rightarrow d_{xz/yz}$ |

After establishing the origin of the spin Hamiltonian parameters at the equilibrium structure, the next step is to examine how each individual vibrational modes perturb them. Modes that most strongly modulate the key electronic transitions identified above (if translated to the orbital representation, the energy level of $d_{xy}$ or $d_{xz/yz}$) are expected to yield the largest spin-phonon coupling parameters.

The vibrational modes can still be grouped by first-coordination sphere motions (symmetric/asymmetric bending and stretching), but in this complex, the ligand scaffold couples with these motions. For example, two low-lying twisting modes (Figure S18) represent in-phase and out-of-phase combinations with the naphthalene ring. The in-phase mode, where the first coordination sphere moves together with the ligand, has a much lower frequency and smaller displacement of the first-coordination sphere compared to the out-of-phase mode.

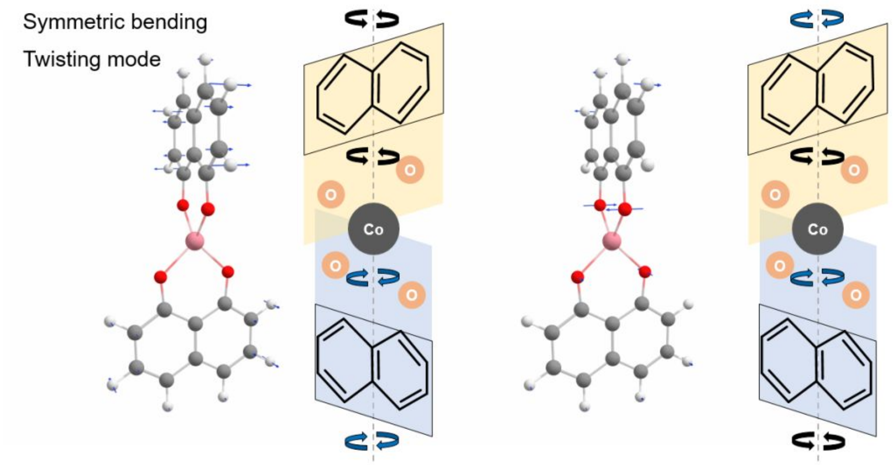


**Figure S18.** Twisting mode: The in-phase and out-of-phase combination between the first-coordination sphere and ligand scaffold.

The symmetric bending modes provide the dominant contribution to spin-phonon coupling, as they significantly modulate the energy levels of $d_{xy}$ and $d_{xz/yz}$. The scissoring mode modulates the *D* value, while the twisting mode also modulates rhombicity. The symmetric stretch also contributes by scaling the overall ZFS, although its effect is smaller.

Asymmetric bending and stretching can also significantly influence the magnetic properties. However, because of the symmetry, their effect on the spin Hamiltonian parameters is typically parabolic with respect to the normal coordinate. As illustrated in Figure S19, positive and negative displacements induce mirror-like magnetic responses, such that the linear spin-phonon coupling term vanishes while the quadratic term remains finite. If the equilibrium geometry deviates from strict axial symmetry, these modes can generate sizable linear couplings. In this system, asymmetric bending and stretching along z are difficult to separate and are therefore grouped as asymmetric stretching along z. These modes are only asymmetric modes that yield nonzero $E^{\alpha}$, as their displacements are not mirror symmetric along z.

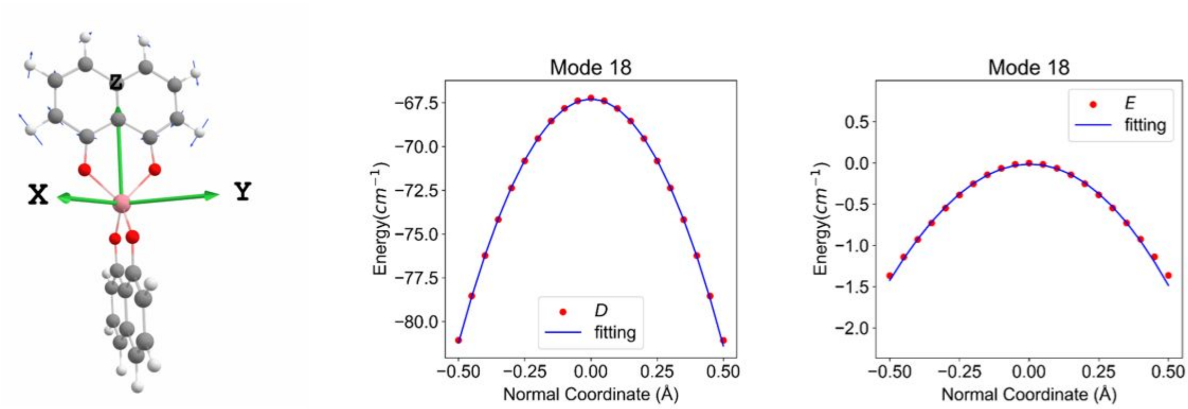


**Figure S19.** (left) Asymmetric stretching (in-phase) along x/y direction. (right) ZFS parameters as the functions of the normal coordinate.

Table S3 summarizes the spin-phonon coupling parameters of modes excluding ligand-local C-C stretches and C-H bending/stretching, which are irrelevant here. In this axial system, there's no rotation of the principal axes of D-tensor. The coupling magnitudes are on the order of 1 $cm^{-1}$, smaller than those in $[CoCl_4]^{2-}$ model. This reduction likely reflects two contributions: (1) oxygen-based ligand are slightly stronger ligands which causes larger ligand field splitting, thus modulation in the first coordination sphere leads to weaker response in the magnetic properties which scales inversely with the excitation energies; (2) the first-coordination sphere displacements across the normal modes get "diluted" with the larger ligand framework, as they have to couple with the vibrational motions of the ligand scaffold.

**Table S3.** Spin-phonon coupling parameters of vibrational modes except pure ligand motions. Mode counting starts from 0, with the first six modes corresponding to overall molecular translations and rotations. For all these modes, in-phase and out-of-phase denote the relative combination pattern with motions of the ligand scaffold.

| Mode | Translation of mode | Frequency $(cm^{-1})$ | $\|D^{\alpha}\|$ $(cm^{-1})$ | $\|E^{\alpha}\|$ $(cm^{-1})$ |
|---|---|---|---|---|
| 6, 7 | Wiggle of the whole structure | 20 | 0.00 | 0.00 |
| 8 | Twisting mode<br>In-phase | 25 | 0.00 | **0.54** |
| 9, 10 | Asymmetric bending along x/y<br>In-phase | 87 | 0.00 | 0.00 |
| 11 | Rotation of the whole $[CoO_4]$ unit | 88 | 0.00 | 0.00 |
| 12 | Twisting mode<br>Out-of-phase | 110 | 0.00 | **1.07** |
| 13 | Scissoring mode<br>In-phase | 134 | **5.71** | 0.00 |
| 18, 19 | Asymmetric stretching along x/y<br>In-phase | 260 | 0.00 | 0.00 |
| 20 | Asymmetric stretching along z<br>In-phase | 271 | 0.00 | **0.60** |
| 21, 22 | Asymmetric bending along x/y<br>Out-of-phase | 275 | 0.00 | 0.00 |
| 23 | Scissoring mode | 361 | **2.57** | 0.00 |

| | | | | |
|---|---|---|---|---|
| | Out-of-phase | | | |
| 24 | Asymmetric stretching along z<br>Out-of-phase | 381 | 0.00 | **0.41** |
| 25, 26 | Asymmetric bending along x/y<br>Out-of-phase | 411 | 0.00 | 0.00 |
| 29 | Symmetric stretching<br>In-phase | 499 | **0.23** | 0.00 |
| 37, 38 | Asymmetric stretching along x/y<br>Out-of-phase | 603 | 0.00 | 0.00 |
| 43 | Symmetric stretching<br>Out-of-phase | 712 | **1.24** | 0.00 |
| 44 | Asymmetric stretching along z<br>Out-of-phase | 714 | 0.00 | **0.40** |

## Theoretical Model of complex 1

With the foundation of the two preceding models, we now turn to the analysis of complex **1**. Like $[Co(ndh)_2]^{2-}$, complex **1** features an "elongated" tetrahedral coordination geometry. The key difference lies in its larger ligand scaffold, which contains two distinct donor sites, one oxygen-based and one nitrogen-based. This asymmetry introduces slight rhombicity to the system. Complex **1** retains easy-plane magnetization, with three low-lying excited states that primarily govern its spin Hamiltonian parameters, as illustrated in Figure S20. Details of all ten quartet roots are given in Table S4.

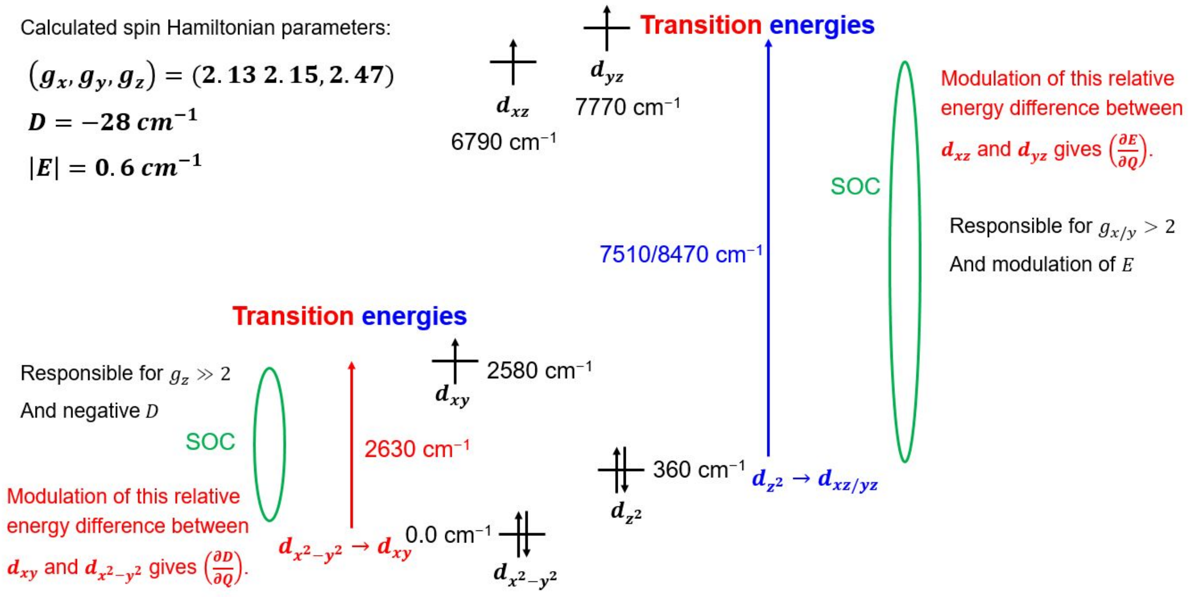


**Figure S20.** AILFT orbital energies (black) and key excited states with transition energies (red and blue) of complex **1**.

One may wonder why the lowest excitation is not the $d_{z2} \rightarrow d_{xy}$ as their AILFT orbital energy levels are closest. The reason is, the excitation energy cannot be equated directly with orbital energy differences. Electron-electron exchange and repulsion also play significant role. Moreover, each electronic state (root) is not described by a single configuration but rather by a mixture of configurations. The in-phase and out-of-phase combinations of these contributing configurations can further lead to markedly different valence excitation energies.

**Table S4.** Calculated energies and dominant configuration state function (CSF) weights (larger than 0.2) of each root. The orbital sequence is displayed as $d_{z2}$, $d_{xz}$, $d_{yz}$, $d_{x2-y2}$, $d_{xy}$, 21121 denotes the occupation number of that CSF is $(d_{z2})^2(d_{xz})^1(d_{yz})^1 (d_{x2-y2})^2(d_{xy})^1$.

| Root | Energy ($cm^{-1}$) | | weight | CSF | Translation |
|---|---|---|---|---|---|
| | CASSCF | NEVPT2 | | | |
| 0 | 0 | 0 | 0.87071 | 21121 | Ground state |
| 1 | 2190 | 2630 | 0.81311 | 21112 | Single excitation: $d_{x2-y2} \rightarrow d_{xy}$ |
| 2 | 6010 | 7510 | 0.80879 | 11221 | Single excitation: $d_{z2} \rightarrow d_{yz}$ |
| 3 | 6770 | 8470 | 0.69710 | 12121 | Single excitation: $d_{z2} \rightarrow d_{xz}$ |
| 4 | 7540 | 9000 | 0.30282<br>0.24527<br>0.22243 | 12211<br>11122<br>12112 | Double excitation:<br>$d_{x2-y2} \rightarrow d_{xy}$<br>$d_{z2} \rightarrow d_{xz/yz}$<br>and<br>Single excitation:<br>$d_{z2} \rightarrow d_{xy}$ |
| 5 | 7890 | 9900 | 0.76875<br>0.20743 | 11212<br>22111 | Double excitation:<br>$d_{x2-y2} \rightarrow d_{xy}$<br>$d_{z2} \rightarrow d_{xz/yz}$ |
| 6 | 9980 | 12020 | 0.39466<br>0.26514 | 12112<br>11122 | Single excitation:<br>$d_{z2} \rightarrow d_{xy}$<br>and<br>Double excitation:<br>$d_{x2-y2} \rightarrow d_{xy}$<br>$d_{z2} \rightarrow d_{xz/yz}$ |
| 7 | 22480 | 21000 | 0.44164<br>0.40919 | 11122<br>12211 | Single excitation:<br>$d_{z2} \rightarrow d_{xy}$<br>and<br>Double excitation:<br>$d_{x2-y2} \rightarrow d_{xy}$<br>$d_{z2} \rightarrow d_{xz/yz}$ |
| 8 | 25160 | 23700 | 0.57423 | 22111 | Single excitation: $d_{x2-y2} \rightarrow d_{xz}$ |
| 9 | 25380 | 24300 | 0.51954 | 21211 | Single excitation: $d_{x2-y2} \rightarrow d_{yz}$ |

Because complex **1** is only near-axial, the strict axial symmetry present in $[Co(ndh)_2]^{2-}$ is already broken. Consequently, the asymmetric bending and stretching modes now exhibit nonzero first-order spin-phonon couplings. However, the reduced symmetry and asymmetric ligand framework cause these modes to mix further, making it difficult to attribute displacements of the metal center cleanly to pure x, y, or z directions. Although in principle such modes could induce rotation of the principal axes of the D-tensor, in practice this effect is negligible, at least in complex **1**. Thus, the earlier approximation, that the principal axes remain essentially fixed, remains valid. Table S5 summarizes the calculated spin-phonon coupling parameters for vibrational modes below 350 $cm^{-1}$. The stretching vibrational modes lie at higher frequencies, and only bending modes are observed in this low-frequency region.

As shown in Table S5, the typical magnitude of $E^{\alpha}$ is about 0.1 $cm^{-1}$, whereas $D^{\alpha}$ ranges from 0.1-1 $cm^{-1}$. Experimentally, the energy splitting between the two Kramers doublets is 47 $cm^{-1}$, and the calculated splitting of 56 $cm^{-1}$ is in good agreement. We note that low-frequency vibrational modes may differ between the gas phase and a crystal environment, potentially altering couplings and frequencies. Therefore, we performed a sequential analysis of $[CoCl_4]^{2-}$, $[Co(ndh)_2]^{2-}$, and complex **1** to elucidate how each type of symmetric and asymmetric bending and stretching mode contributes to the corresponding spin-phonon coupling. Nevertheless, even within a reasonable error margin, the vibrational mode most likely responsible for the second-derivative feature in the experimental field-division spectra is the twisting mode. The vibrational frequency and motion within the first-coordination sphere are unlikely to differ qualitatively between the gas phase and the crystal. Among the low-frequency modes below 150 $cm^{-1}$, the twisting mode induces the largest modulation of the $d_{xz}/d_{yz}$ energy levels and thus exhibits the largest $E^{\alpha}$. This assignment is further supported by its weak IR intensity, as this symmetric bending is IR forbidden under axial symmetry. With slight rhombicity, this vibrational transition is weakly IR allowed, which rationalizes the comparable strengths of the phonon absorption and EPR transitions observed in the experimental spectra.

Additionally, as CASSCF wavefunctions are relatively ionic and tend to overestimate SOC strength, they naturally overestimate spin Hamiltonian parameters even with NEVPT2 correction and consequently the modulation of these spin Hamiltonian parameters (the spin-phonon coupling parameters). If we take this effect into consideration, the calculated $E^{\alpha}$ of 0.28 $cm^{-1}$ is in close agreement with the experimental determined value of 0.16 $cm^{-1}$.

**Table S5.** Spin-phonon coupling parameters of vibrational modes below 350 $cm^{-1}$. Mode counting starts from 0, with the first six modes corresponding to overall molecular translations and rotations. Here "---" in the translation of mode denotes that this mode is a mixture of "wiggle of the whole structure" and "asymmetric bending".

| Mode | Translation of mode | Frequency $(cm^{-1})$ | $\|D^{\alpha}\|$ $(cm^{-1})$ | $\|E^{\alpha}\|$ $(cm^{-1})$ |
|---|---|---|---|---|
| 6 | --- | 13 | 0.02 | 0.08 |
| 7 | --- | 17 | 0.04 | 0.12 |
| 8 | --- | 31 | 0.14 | 0.11 |
| 9 | --- | 38 | 0.13 | 0.02 |
| 10 | --- | 44 | 0.03 | 0.06 |
| 11 | Rotation of the [$CoO_2N_2$] unit | 58 | 0.08 | 0.03 |
| 12 | Twisting mode<br>In-phase | 60 | 0.24 | 0.28 |
| 13 | --- | 67 | 0.57 | 0.03 |
| 14 | --- | 77 | 0.13 | 0.12 |
| 15 | --- | 107 | 0.60 | 0.04 |
| 16 | Scissoring mode + asymmetric bending<br>In-phase | 108 | 1.62 | 0.21 |
| 17 | Scissoring mode<br>In-phase | 122 | 1.99 | 0.07 |
| 18 | --- | 145 | 0.33 | 0.00 |
| 19 | --- | 155 | 0.10 | 0.38 |
| 20 | --- | 196 | 0.03 | 0.08 |
| 21 | --- | 211 | 0.46 | 0.01 |
| 22 | --- | 229 | 0.02 | 0.16 |
| 23 | --- | 235 | 0.60 | 0.06 |
| 24 | --- | 260 | 0.40 | 0.06 |
| 25 | --- | 265 | 1.10 | 0.00 |
| 26 | --- | 277 | 0.30 | 0.01 |
| 27 | --- | 297 | 0.57 | 0.02 |
| 28 | --- | 323 | 0.01 | 0.01 |

# Molecular Coordinates

## $[CoCl_4]^{2-}$

| | | | |
|---|---|---|---|
| Co | 0.0000000 | 0.0000000 | 0.0000000 |
| Cl | -1.3551608 | 1.3551608 | 1.3551608 |
| Cl | 1.3551608 | -1.3551608 | 1.3551608 |
| Cl | 1.3551608 | 1.3551608 | -1.3551608 |
| Cl | -1.3551608 | -1.3551608 | -1.3551608 |

## $[Co(ndh)_2]^{2-}$

| | | | |
|---|---|---|---|
| Co | 0.000000000000 | 0.000000000000 | 0.000000000000 |
| O | 1.000100961835 | 1.000100961835 | -1.327552000000 |
| O | -1.000100961835 | -1.000100961835 | -1.327552000000 |
| O | 1.000100961835 | -1.000100961835 | 1.327552000000 |
| O | -1.000100961835 | 1.000100961835 | 1.327552000000 |
| C | 0.904927040981 | 0.904920623454 | -2.627066000000 |
| C | 1.743808471128 | 1.743795076188 | -3.405995000000 |
| C | 1.722392312372 | 1.722373072871 | -4.805513000000 |
| C | 0.871071246381 | 0.871061142093 | -5.493519000000 |
| C | 0.000000000000 | 0.000000000000 | -4.777121000000 |
| C | 0.000000000000 | 0.000000000000 | -3.328292000000 |
| C | -0.904927040981 | -0.904920623454 | -2.627066000000 |
| C | -1.743808471128 | -1.743795076188 | -3.405995000000 |
| C | -1.722392312372 | -1.722373072871 | -4.805513000000 |
| C | -0.871071246381 | -0.871061142093 | -5.493519000000 |
| H | 2.412509106448 | 2.412493295977 | -2.857694000000 |
| H | 2.391487614987 | 2.391458387900 | -5.362506000000 |
| H | 0.851048798195 | 0.851037135859 | -6.588505000000 |
| H | -0.851048798195 | -0.851037135859 | -6.588505000000 |
| H | -2.391487614987 | -2.391458387900 | -5.362506000000 |
| H | -2.412509106448 | -2.412493295977 | -2.857694000000 |
| H | -0.851037135859 | 0.851048798195 | 6.588505000000 |
| C | -0.871061142093 | 0.871071246381 | 5.493519000000 |
| C | -1.722373072871 | 1.722392312372 | 4.805513000000 |
| C | -1.743795076188 | 1.743808471128 | 3.405995000000 |
| C | -0.904920623454 | 0.904927040981 | 2.627066000000 |
| C | 0.000000000000 | 0.000000000000 | 3.328292000000 |
| C | 0.000000000000 | 0.000000000000 | 4.777121000000 |
| C | 0.871061142093 | -0.871071246381 | 5.493519000000 |
| C | 1.722373072871 | -1.722392312372 | 4.805513000000 |
| C | 1.743795076188 | -1.743808471128 | 3.405995000000 |
| C | 0.904920623454 | -0.904927040981 | 2.627066000000 |
| H | -2.391458387900 | 2.391487614987 | 5.362506000000 |
| H | -2.412493295977 | 2.412509106448 | 2.857694000000 |
| H | 2.412493295977 | -2.412509106448 | 2.857694000000 |
| H | 2.391458387900 | -2.391487614987 | 5.362506000000 |
| H | 0.851037135859 | -0.851048798195 | 6.588505000000 |

Complex **1**

| | | | |
|---|---|---|---|
| Co | 0.000000000000 | 0.000000000000 | 0.000000000000 |
| O | -0.912817133124 | 0.909205871610 | -1.401887903579 |
| O | 0.806198038613 | 0.984941442090 | 1.422674405675 |
| N | 1.261657258834 | -0.998901027396 | -1.164611862700 |
| N | -1.191511804983 | -1.110742095009 | 1.135654490598 |
| C | 0.538627851711 | 0.854972624909 | 2.687273130414 |
| C | 1.234092254703 | 1.672648046239 | 3.613844238297 |
| C | -0.414243248211 | -0.080007230016 | 3.218574864094 |
| C | 1.014193364015 | 1.577237209265 | 4.968560708589 |
| H | 1.948181454238 | 2.378743476636 | 3.207453301324 |
| C | -0.607584270076 | -0.145634415443 | 4.620720145777 |
| C | 0.084297288036 | 0.659527850008 | 5.491300355128 |
| H | 1.567272443732 | 2.222156169153 | 5.643301085038 |
| H | -1.332024111960 | -0.860267111774 | 4.999808023777 |
| H | -0.082008189914 | 0.592016614212 | 6.558953812229 |
| C | -0.601641807364 | 0.876915159554 | -2.659771804504 |
| C | 0.456522538741 | 0.073293742454 | -3.211986121720 |
| C | -1.349878943822 | 1.675209826572 | -3.563621289585 |
| C | 0.704151455022 | 0.121808988530 | -4.607467301331 |
| C | -1.076553738086 | 1.689827586785 | -4.910791140903 |
| H | -2.147752719490 | 2.275332711561 | -3.143438913592 |
| C | -0.037492163688 | 0.908209741435 | -5.452265611548 |
| H | 1.509226664995 | -0.491686073057 | -5.000965289998 |
| H | -1.672075955466 | 2.315853098390 | -5.566973748590 |
| H | 0.169424785202 | 0.928111855733 | -6.514765775303 |
| C | 1.286998180229 | -0.799747736886 | -2.455940672893 |
| H | 2.029229883128 | -1.354844789635 | -3.034324080394 |
| C | 2.094985736461 | -1.996757050005 | -0.602891938690 |
| C | 2.648325549339 | -1.766973820306 | 0.659649589542 |
| C | 2.321806265341 | -3.218332269433 | -1.244677628695 |
| C | 3.440141098192 | -2.737929153656 | 1.257194062718 |
| H | 2.459750718986 | -0.818621608378 | 1.149593244250 |
| C | 3.114252528784 | -4.185250622886 | -0.637352434972 |
| H | 1.851934107701 | -3.422134471340 | -2.200234422452 |
| C | 3.679052477672 | -3.949221307436 | 0.612255186603 |
| H | 3.871446362924 | -2.546700473974 | 2.232892689539 |
| H | 3.276928913638 | -5.133219315450 | -1.137296873779 |
| H | 4.290662806276 | -4.708175221211 | 1.085383792223 |
| C | -1.204207290708 | -0.968745090120 | 2.432852196313 |
| H | -1.884246184881 | -1.609944456447 | 2.998524467017 |
| C | -2.118608592340 | -2.004499479193 | 0.542927474384 |
| C | -3.447043659472 | -2.071634371950 | 0.971582036010 |
| C | -1.697293434148 | -2.801508182551 | -0.523425453334 |
| C | -4.333023300790 | -2.946068055582 | 0.353876585907 |
| C | -2.587367403862 | -3.675206901722 | -1.132421428353 |
| C | -3.907502345845 | -3.753805660612 | -0.696233668153 |
| H | -3.789877980261 | -1.412633338928 | 1.760729345854 |
| H | -0.667867525953 | -2.739052508175 | -0.852469507225 |
| H | -5.364397271443 | -2.983619172058 | 0.685288722879 |
| H | -2.248220927962 | -4.296795327913 | -1.953000753313 |
| H | -4.602451452391 | -4.429991141570 | -1.179676914925 |
